# The Negative Relationship between Reasoning and Religiosity Is Underpinned by a Bias for Intuitive Responses Specifically When Intuition and Logic Are in Conflict

**DOI:** 10.3389/fpsyg.2017.02191

**Published:** 2017-12-19

**Authors:** Richard E. Daws, Adam Hampshire

**Affiliations:** The Computational, Cognitive and Clinical Neuroimaging Laboratory (C^*3*^NL), Imperial College London, London, United Kingdom

**Keywords:** religiosity, reasoning, intelligence, cognition, conflict detection

## Abstract

It is well established that religiosity correlates inversely with intelligence. A prominent hypothesis states that this correlation reflects behavioral biases toward intuitive problem solving, which causes errors when intuition conflicts with reasoning. We tested predictions of this hypothesis by analyzing data from two large-scale Internet-cohort studies (combined *N* = 63,235). We report that atheists surpass religious individuals in terms of reasoning but not working-memory performance. The religiosity effect is robust across sociodemographic factors including age, education and country of origin. It varies significantly across religions and this co-occurs with substantial cross-group differences in religious dogmatism. Critically, the religiosity effect is strongest for tasks that explicitly manipulate conflict; more specifically, atheists outperform the most dogmatic religious group by a substantial margin (0.6 standard deviations) during a color-word conflict task but not during a challenging matrix-reasoning task. These results support the hypothesis that behavioral biases rather than impaired general intelligence underlie the religiosity effect.

## Introduction

The relationship between religiosity and intelligence has been an important topic amongst scientists and the public for some time (Harris, 2004; Dennett, 2006; Hitchens, 2007; Dawkins, 2008). Early evidence from the twentieth century suggested that religiosity and intelligence negatively correlated amongst college students (Howells, 1928; Sinclair, 1928). Subsequently, Argyle (1958) concluded that intelligent students are less likely to be religious. More recently, scientists have shown a striking paucity of religious belief (Ecklund et al., 2016), particularly within the elites of the National Academy of Sciences (Larson and Witham, 1998) and the Royal Society (Stirrat and Cornwell, 2013).

Psychometric population studies have now firmly established that religiosity influences cognitive style (Shenhav et al., 2012), and that religiosity and intelligence negatively correlate (Verhage, 1964; Pargament et al., 1998; Nyborg, 2009; Gervais and Norenzayan, 2012; Pennycook et al., 2013, 2014; Razmyar and Reeve, 2013; Zuckerman et al., 2013). Furthermore, it has been reported that IQ and disbelief in God correlate at *r* = 0.60 across 137 countries (Lynn et al., 2009).

The cognitive sciences are establishing a mechanistic understanding of the religiosity effect. For example, it has been seen that religious background modulates visual attention (Colzato et al., 2008). Lesion studies have demonstrated that ventro-medial prefrontal cortex lesion patients have elevated scores of religious fundamentalism (Asp et al., 2012). Experimental studies have demsontrated that increases in religious fundamentalism relate to increases in memory recall accuracy and higher rates of false-positives in a memory task (Galen et al., 2009). Religious fundamentalism has also shown modest positive correlations with life satisfaction (Carlucci et al., 2015) and negative correlations with cognitive flexibility (Zhong et al., 2017) and openness (Saroglou, 2002; Carlucci et al., 2011, 2015).

Dual-process models (Evans, 2008) assert that cognition is composed of intuitive and logical information processing. Individual differences in cognitive style have been related to the propensity to engage logical processes during problem solving (Stanovich and West, 1998). Meanwhile, recent experimental evidence has demonstrated a link between religiosity and cognitive style (Gervais and Norenzayan, 2012; Pennycook et al., 2014). From this, a prominent hypothesis has emerged which suggests that the religiosity effect is underpinned by cognitive-behavioral biases that cause poorer detection of situations in which intuition and logic are in conflict (Pennycook et al., 2014). Put simply, religious individuals are less likely to engage logical processes and be less efficient at detecting reasoning conflicts; therefore, they are more likely to take intuitive answers at face value and this impairs performance on intelligence tests. More broadly, from the perspective of this “dual-process” hypothesis, religious cognition is facilitated and hallmarked by intuitive decision making (Norenzayan and Gervais, 2013; Morgan, 2014; Oviedo, 2015).

It can be predicted from this hypothesis that the religiosity effect should be particularly disadvantageous for handling problems with counterintuitive answers; however, as a cognitive-behavioral bias, rather than reduced cognitive capacity *per se*, it follows that religiosity may not affect all tasks that involve reasoning. Reasoning tasks without intuitively obvious but logically correct answers may engage religious individual's latent ability to resolve complicated problems.

Here, we apply a novel combination of analyses to data from two Internet-cohort studies with detailed sociodemographic questionnaires and performance data from multiple cognitive tasks. Critically, these cohorts are large enough for the religiosity effect to be reliably examined in relation to, and while factoring out, a range of potentially confounding sociodemographic factors.

In study 1, we test four predictions of the dual-process hypothesis. (1) The religiosity effect should be greatest for reasoning latent variables as resolved via factor analysis. (2) The religiosity effect should be greatest for reasoning tasks designed to involve conflict resolution. (3) The religiosity effect should be in addition to, and not dependent on, other sociodemographic variables. (4) The pattern of the religiosity effect across tasks should differ qualitatively from those observed for other sociodemographic factors relating to latent reasoning ability.

In study 2, we replicate the findings of study 1 and test the further predictions that religious dogmatism mediates the religiosity-reasoning relationship at the levels of individuals (5) and religious groups (6). Finally, we test whether conversion to, or apostasy from, a religious group predicts cognitive performance (7).

## Materials and methods

The cognitive tasks were all designed/adapted and programmed in Adobe Flex 3 for the Internet. The tasks were based on classical paradigms from the cognitive neuroscience literature to measure planning, reasoning, attention, and working memory abilities. The entire battery of tasks took ~30 min to complete, with each task calculating one outcome measure (Full descriptions of all task designs are reported in Supplementary Materials 2). The tasks were presented in fixed sequence on a custom Internet server. A detailed demographic assessment was conducted after completion of the task battery and this also was programmed using Adobe Flex. The server for study 1 was programmed in ASP. The server for study 2 was programmed in Ruby on Rails. The data for study 1 were collected via the Internet between September and December 2010. The experiment URL was originally advertised in a *New Scientist* feature, on the *Discovery Channel* website, in the *Daily Telegraph*, and on social networking websites including Facebook and Twitter (for further details please refer to Hampshire et al., 2012a). Study 2 was run in a similar manner, but with a slightly different sub-set of tasks. Data were collected in the first 4 months of 2013 with advertisement through a press release associated with another article that was published with data from the first study. Ethical approval for the study protocol was awarded by the Cambridge Psychology Research Ethics Committes (2010.62) and the University of Western Ontario Health Sciences Research Ethics Board (103472) for study 1 and 2 respectively. All subjects gave informed consent in accordance with the Declaration of Helsinki prior to being able to access the cognitive and demographic assessment stages.

Statistical analyses were conducted in Matlab (R2015b, www.mathworks.com), unless otherwise stated. Data from both studies were preprocessed using the following steps. Participants with ages below 15 or above 90 and subjects with nonsensical responses to any questionnaire question were excluded case-wise (see Hampshire et al., 2012a for details). The cognitive data were standardized task-wise by subtracting the population mean to center scores around zero and division by the population standard deviation to ensure unit deviation. A wide filter of scores >5 SDs from the mean on any task were excluded case-wise to remove any machine errors. Sociodemographic confounds including Age, Level of Education and Country of Origin were controlled for by modeling them as main effects in a Generalized Linear Model and extracting the resulting residuals using the SPSS V22 (Supplementary Materials 1).

Study 1 included 44,780 individuals; 12,576 reported themselves to be religious (Mean age = 31.38, SD = 12.02), 14,018 agnostic (Mean age = 30.12, SD = 10.99) and 18,186 atheist (Mean age = 29.98, SD = 11.26). Study 2 included 18,455 individuals; 10,876 reported themselves to be religious (Mean age = 34.02, SD = 14.26), 2,612 agnostic (Mean age = 30.44, SD = 12.31) and 4,967 atheist (Mean age = 29.73, SD = 11.86). When analyzing the religious sub-groups, 3 groups were excluded due to low sample sizes (Religious Group 6 = 93, Religious Group 7 = 51, Religious Group 8 = 10). Sociodemographic variables are reported in detail in the Supplementary Materials (Supplementary Tables 1–3, N.B. We have previously demonstrated that gender does not have a significant effect on cognitive performance in Owen et al., 2010; Hampshire et al., 2012a).

**Figure 1:**
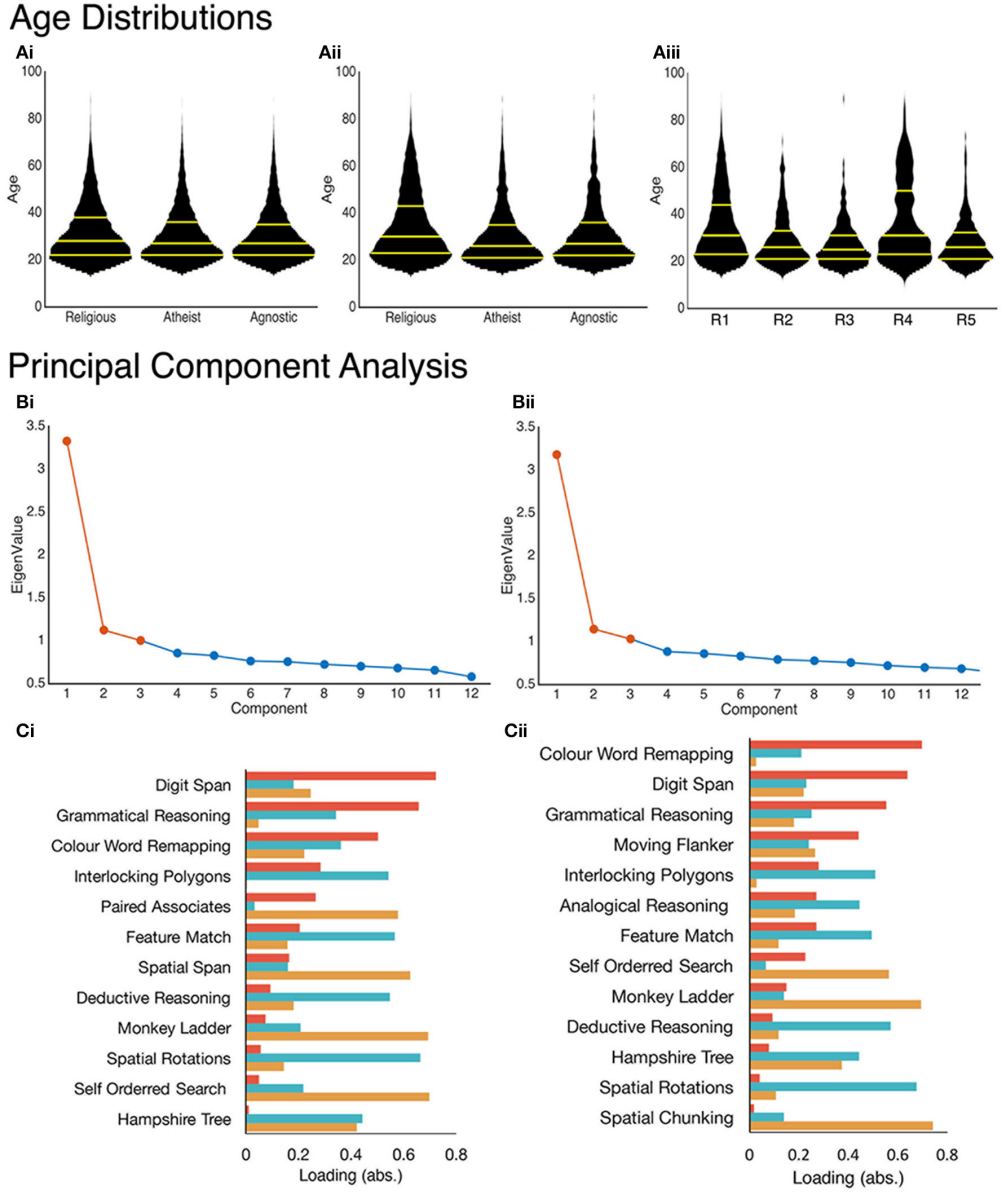
**(A)** Age distributions plotted for each group in study 1 **(Ai)** and for study 2 **(Aii,Aiii)**. Yellow lines indicate the 25, 50, and 75th percentiles within each groups distributions (bottom-to-top). PCA produced 3-component solutions (eigenvalues >1) for both study's. **Bi,Bii**) Scree plots with those components extracted for calculating individual factor scores highlighted in orange. **Ci,Cii**) Absolute loadings calculated (Verbal Reasoning = red, Reasoning = blue, Working Memory = orange) using a Varimax rotation plotted for each task (ranked by Verbal Reasoning loading).

Latent variables were estimated separately from the studies 1 and 2 performance data in a data-driven manner using principal component analysis (PCA). Following the Kaiser convention there were 3 significant components (Eigenvalue, EV ≥ 1) in both cases. In Study 1, the first three unrotated components explained ~45% of the population variance in performance (C1 = 27.733%, EV = 3.328, C2 = 9.359%, EV = 1.123, C3 = 8.355%, EV = 1.002). In study 2, the first three unrotated explained ~41% of the total variance (C1 = 24.469%, EV = 3.181, C2 = 8.812%, EV = 1.146, C3 = 7.934%, EV = 1.031) (Figure 1B). When orthogonal rotation was applied using the varimax algorithm (Rotated variance explained: Study 1, C1 = 17.154, C2 = 16.245, C3 = 12.047; Study 2, C1 = 14.904, C2 = 13.170, C3 = 13.140), the resultant task-component loadings were simple and interpretable (Figure 1C, Supplementary Tables 7, 8). They were also qualitatively similar across the two studies, despite differences in the exact composition of the batteries. For example, the Colour Word Remapping (CWR), a variant of the Stroop task) and Grammatical Reasoning loaded onto a *Verbal Reasoning* component, the Deductive Reasoning and Spatial Rotations tasks loaded onto a more general *Reasoning* component and the Paired Associate Learning (PAL), Spatial Span and Self-Ordered Search tasks loaded onto a *Working Memory* component. Notably, the Grammatical Reasoning and CWR tasks loaded more heavily onto the Reasoning component in study 1 and more heavily on the Verbal Reasoning component in study 2, which likely reflects differences in the exact compositions of the two testing batteries. A quantitative comparison of the 10 task-component loadings that were common across studies 1 and 2 showed extremely high correlation (Verbal Reasoning: *r* = 0.983, *p* < 0.001; Reasoning: *r* = 0.978, *p* < 0.001; Working Memory: *r* = 0.923, *p* < 0.001). It was suggested during the review process that an alternative dimensionality reduction technique, Principal Axis Factoring (PAF), be applied to the data instead of PCA. A comparison of PCA and PAF was conducted using the data from Study 1 (In both cases we followed the Kaiser convention and applied varimax rotation). This analysis demonstrated that PCA provided a much more interpretable latent structure (Supplementary Figure 1) and explained substantially more of the total variance (~45 and ~28%, respectively). From this comparison PCA was deemed as a more appropriate method for the present analysis.

It is important to note that analyzing data with extremely large numbers of samples affords very high statistical power, which means that effects of negligible scale can have very low *p*-values; therefore, in studies of this type a better gauge of significance is effect size. Here, we conform to Cohen's notion of effect sizes, whereby an effect of ~0.2 standard deviations (SDs) is small, ~0.5 SDs is medium and ~0.8 SDs is large. All other statistical values from our analyses are reported in the Supplementary Tables and generally are *p* < 0.001 unless otherwise indicated.

There were negligibly scaled but statistically significant differences across the groups in terms of age (Figure 1A), education level, and country of origin (Supplementary Tables 4–6); therefore, these variables were factored out of the performance data prior to the analyses reported below.

## Results

### Determining the scale of the reasoning effect for different latent variables

Component scores were estimated for each individual by regressing task scores onto the rotated component matrix. An “Overall Mean” score was also estimated for each individual by averaging across the three component scores. In order to test prediction (1), these composite scores were analyzed in separate one-way ANOVAs with Religious Class (Religious, Agnostic, Atheist) as the between subject factor for both studies (Supplementary Table 9).

**Figure 2:**
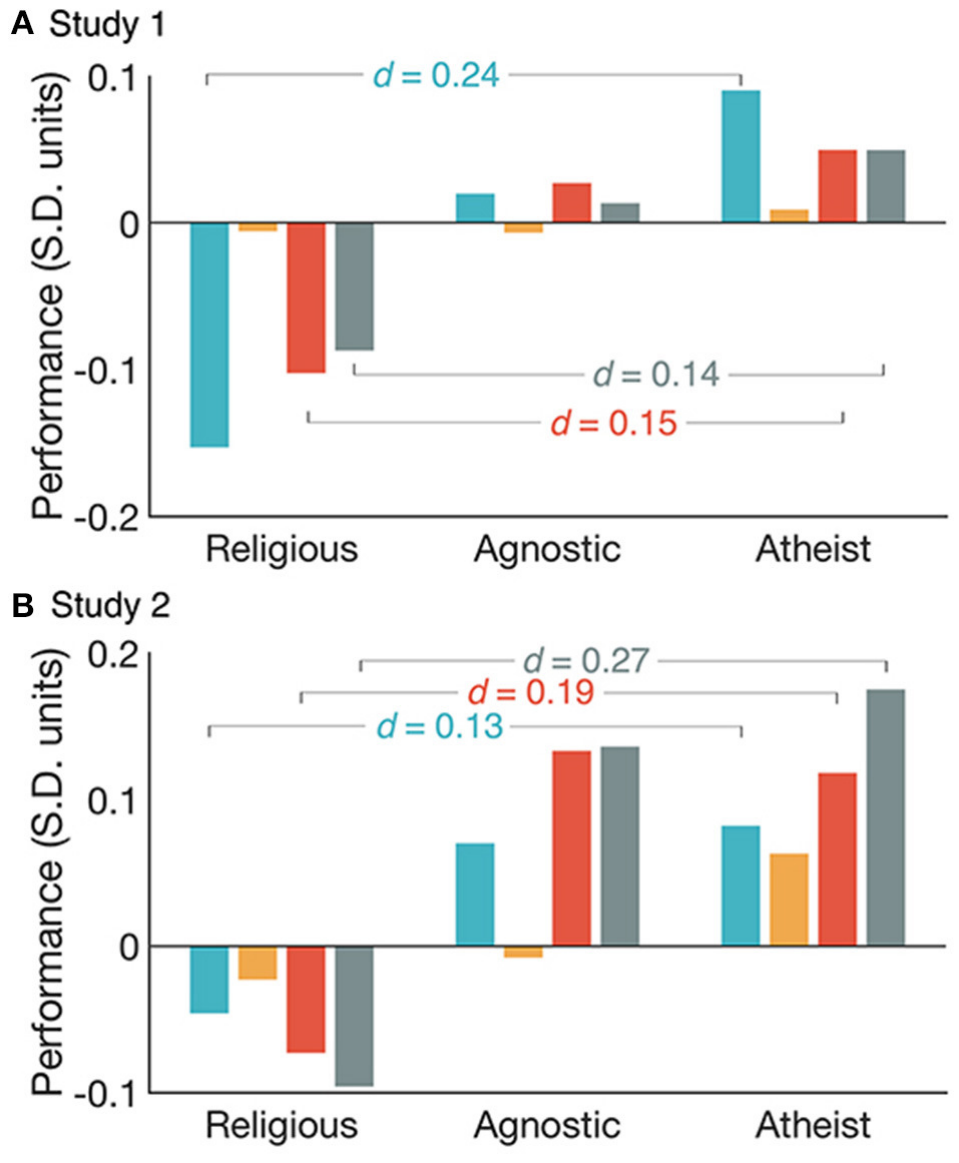
Reasoning (blue), Working Memory (orange), Verbal Reasoning (red) and Overall Mean (gray) component scores in Standard Deviation units (Mean = 0, SD = 1) compared across the Religious, Agnostic and Atheist groups for study 1 **(A)** and study 2 **(B)**. Superimposed Cohens-d values show the magnitude of differences between the Religious and Atheist groups.

Confirming prediction (1), an analysis of effect size demonstrated that the religiosity effects was largest for reasoning latent variables. In study 1, the religious group was outperformed by the agnostic and atheist groups (Figure 2A, Supplementary Tables 9–12) in terms of Reasoning (Agnostic vs. Religious = 0.17 SDs, Atheist vs. Religious = 0.24 SDs), Verbal Reasoning (Agnostic vs. Religious = 0.13 SDs, Atheist vs. Religious = 0.15 SDs) and Overall Mean (Agnostic vs. Religious = 0.10 SDs, Atheist vs. Religious = 0.14 SDs) composite scores. The differences in Working Memory scores were of negligible scale (Agnostic vs. Religious = 0.001 SDs, Atheist vs. Religious = 0.015).

A similar pattern of results was evident for study 2 (Figure 2B, Supplementary Tables 9–12): Reasoning (Agnostic vs. Religious = 0.12 SDs, Atheist vs. Religious = 0.13 SDs) Verbal Reasoning (Agnostic vs. Religious = 0.21 SDs, Atheist vs. Religious = 0.19 SDs) and Overall Mean (Agnostic vs. Religious = 0.23 SDs, Atheist vs. Religious = 0.27 SDs). The effect for Working Memory was again of negligible scale (Agnostic vs. Religious = 0.02 SDs, Atheist vs. Religious = 0.09 SDs). These results confirmed that the religiosity effect is largest for latent variables that underlie the performance of reasoning tasks.

### Determining whether the religiosity effect is more pronounced for reasoning tasks that explicitly manipulate conflict

To test prediction (2), i.e., that the religiosity effect relates to conflict, analyses were conducted focused on performances of individual cognitive tasks. Specifically, several of the cognitive tasks loaded heavily on the reasoning latent variables and were explicitly designed to manipulate conflict. These were, the CWR Task, which in accordance with the classic Stroop paradigm (Stroop, 1935), places color and word mappings in direct conflict. Unlike the traditional Stroop, meaning must be remapped to color and word on every trial, which produces a more pronounced conflict effect (Hampshire et al., 2012a). The Grammatical Reasoning Task involves a rapid sequence of trials that require the relationship as described between two objects (i.e., the square contains the circle) to be parsed and then in half of the trials inverted, i.e., due to inclusion of the word “not” (Baddeley, 1968; Hampshire et al., 2012a,b). The Interlocking Polygons task involves determining whether a line figure presented alone matches another that is presented as part of an overlapping pair, a manipulation designed to cause perceptual conflict (Hampshire et al., 2012a).

Notably, another of the tasks, Deductive Reasoning, also loads heavily onto the reasoning component. This task involves deriving complex relational rules between the colors, numbers and shapes of patterns that are presented in a 3 ^*^ 3 matrix; however, unlike CWR and grammatical reasoning, it has no explicit conflict manipulation because there are no intuitively obvious but erroneous answers (Owen et al., 2010; Hampshire et al., 2012a). Some other tasks are not designed to involve reasoning or conflict, e.g., simple working memory tasks including Digit Span where sequences of numbers must be remembered, Spatial Span where sequences of locations must be remembered, and Monkey ladder where the locations of numbers must be remembered.

The performance data were standardized for each individual task and sociodemographic confounds factored out prior to cross-group analysis. Cognitive data from both studies were examined using a two-way ANOVA with Task as the within subject factor and Religious Class (Religious, Agnostic, Atheist) as the between subject factor. There were significant main effects of Religious Class and a significant interaction of Religious Class ^*^ Task (Supplementary Table 13).

**Figure 3:**
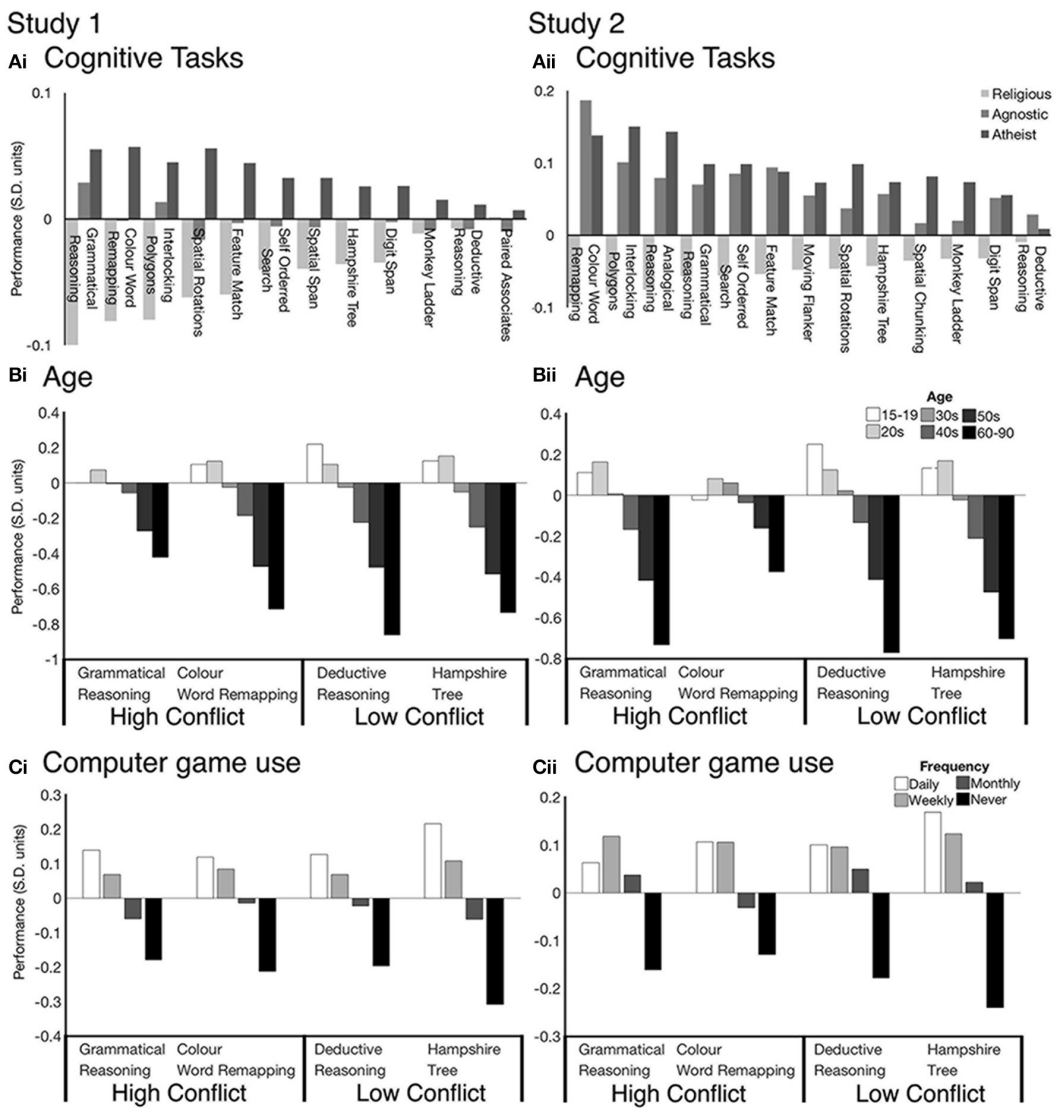
The non-religious groups consistently outperforms the religious group across cognitive tasks in both studies 1 and 2 **(Ai,Aii)**. The largest group effects are seen during cognitive tasks with conflict between intuitive and logical processes (Religious, light gray; Agnostic, gray; Atheist, dark gray). We demonstrate that these effects are specific to religiosity by comparing tasks with high and low cognitive conlfict against alternate demographic variables Age **(Bi,Bii)** and Computer game use **(Ci,Cii)**. Performance scores for all cognitive tasks are in Standard Deviation units (Mean = 0, SD = 1).

Examining the task data showed a consistent trend whereby atheists on average performed numerically better than religious individuals for all tasks, with the agnostics tending to place in between the other two groups. However, the scale of the effect varied substantially across tasks. In support of prediction 2, the largest cross-group effect sizes were observed for tasks that were explicitly designed to manipulate conflict (Figure 3A, Supplementary Table 14). Specifically, in study 1 the largest religious-atheist group differences were for the Grammatical Reasoning (Agnostic vs. Religious = 0.14 SDs, Atheist vs. Religious = 0.17 SDs), CWR (Agnostic vs. Religious = 0.08 SDs, Atheist vs. Religious = 0.14 SDs) and Interlocking Polygons (Agnostic vs. Religious = 0.09 SDs, Atheist vs. religious = 0.13 SDs) tasks. A similar pattern of results was observed in study 2 for the CWR (Agnostic vs. Religious = 0.28 SDs, Atheist vs. Religious = 0.23 SDs) and Interlocking Polygons (Agnostic vs. Religious = 0.18 SDs, Atheist vs. Religious = 0.23 SDs) tasks.

For contrast, the Deductive Reasoning task (study 1: Agnostic vs. Religious = 0.00 SDs, Atheist vs. Religious = 0.02 SDs; study 2: Agnostic vs. Religious = 0.04 SDs, Atheist vs. Religious = 0.01 SDs) and Digit Span task (study 1: Agnostic vs. Religious = 0.03 SDs, Atheist vs. Religious = 0.06 SDs; study 2: Agnostic vs. Religious = 0.08 SDs, Atheist vs. Religious = 0.02 SDs) showed some of the smallest differences in scores between religious and non-religious classes. These findings provide evidence in support of the hypothesis that the religiosity effect relates to conflict as opposed to reasoning ability or intelligence more generally (Pennycook et al., 2014).

### Is the conflict/non-conflict effect specific to religiosity?

One possibility was that the differences in religiosity effect sizes for reasoning tasks may have been generic, e.g., relating to test-retest reliabilities or some other factor that could lead to a general scaling of effect sizes. To rule out this possibility we examined how other demographic variables, which also correlated with the Reasoning latent variables, related to the performance of the individual tasks. These included age and frequency of computer game use. Both age and computer gaming showed similarly scaled relationships with the performance of the conflict (e.g., CWR) are non-conflict (e.g., Deductive Reasoning) tasks (Figures 3B,C).

### Is the religiosity effect contingent on other sociodemographic variables?

**Figure 4:**
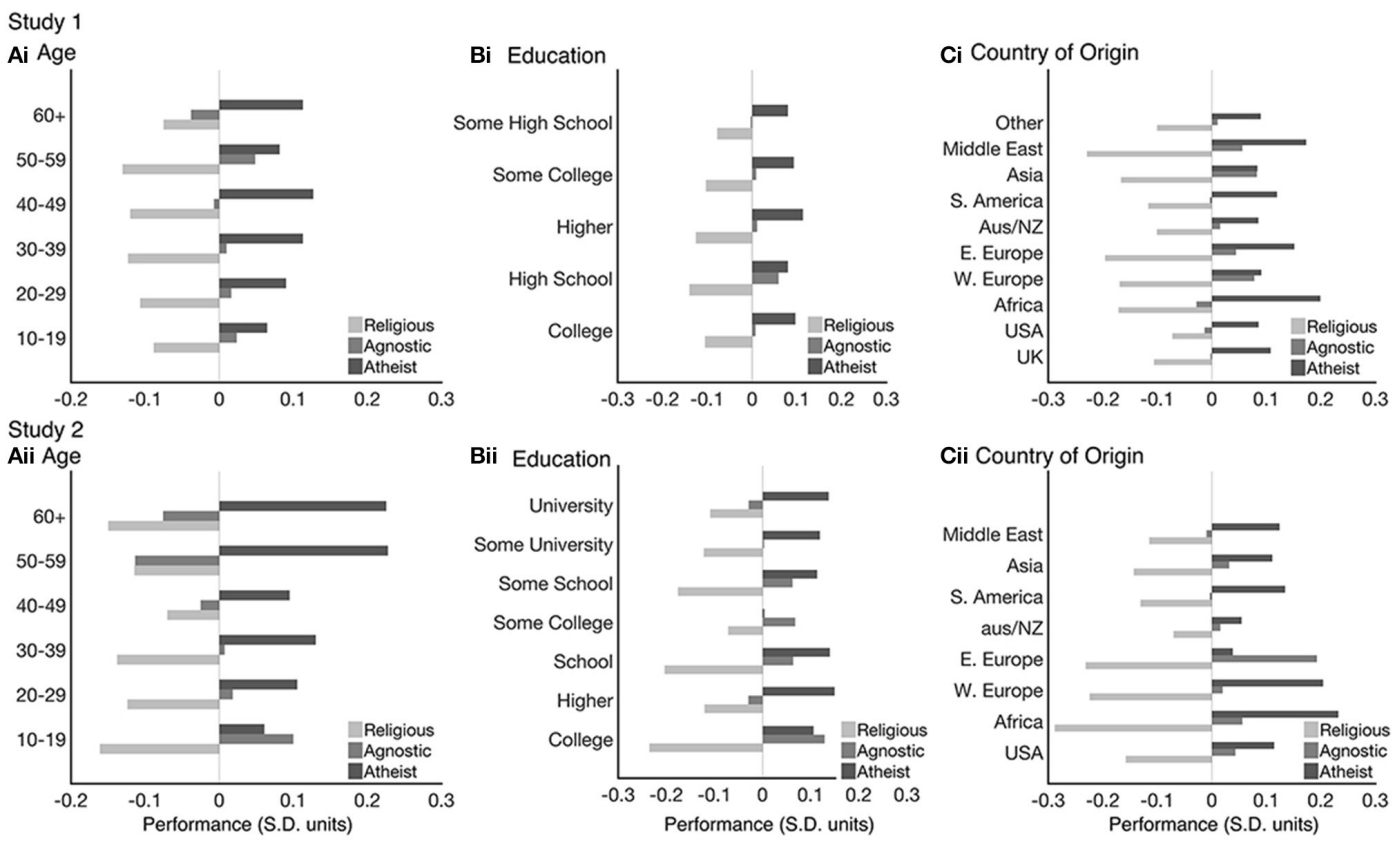
Religious group effects across the lifespan **(Ai,Aii)**, education **(Bi,Bii)** and Country of Origin **(Ci,Cii)** for both study's. These demonstrate how the religiosity effects are not contingent on other sociodemographic variables. Bars represent Overall Mean component score in Standard Deviation units (Mean = 0, SD = 1) for the religious (light gray), agnostic (gray), and Atheist (dark gray) groups.

The analyses thus far factored out potentially confounding sociodemographic variables including age, country of origin and education level. Therefore, these variables did not underlie the religiosity effect. However, the religiosity effect might still have been contingent on those variables (e.g., being evident for older not younger adults). To examine this possibility, further analysis of the with component scores from the religious and non-religious groups were conducted across 6 age bins that covered the adult lifespan from 15 to 90 years. The stability of the Religious Class effects across ages was assessed for both studies using two-way ANOVAs with Overall Mean component score as the dependent variable, and with Religious Class (Religious, Agnostic, Atheist) and Age Group as between subject factors. There was a substantial effect of Age Group. The interaction between Age Group and Religious Class were statistically non-significant (Supplementary Table 15, Figures 4Ai,Aii) whereas the Religious Class main effect was robust and evident across all ages in both studies.

Next, the stability of the religiosity effect was assessed across the Education factor using two-way ANOVAs with Overall Mean component score as the dependent variable and Religious Class (Religious, Agnostic, Atheist) and Level of Education as between subject factors. The interactions between the Religious class and Education factor was statistically non-significant. The main effect of Religious Class was robust and evident across all levels of education (Supplementary Table 15, Figures 4Bi,Bii) in both studies.

Finally, the stability of the religiosity effect was assessed across Country of Origin (i.e., country indivdiuals were born in aggregated by global region) using a two-way ANOVA with Overall Mean component score as the dependent variable and Religious Class (Religious, Agnostic, Atheist) and Country of Origin as between subject factors. The Religious Class main effect was robust and evident across all countries of origin. There was a statistically significant interaction (Supplementary Table 15) for both studies; however, the direction of the effect was evident for all countries of origin (Figures 4Ci,Cii). Together, these analyses confirm that the religiosity effect is highly general, being evident across age group, education level and countries of origin.

### Does religious dogmatism mediate the religiosity-reasoning effect?

**Figure 5:**
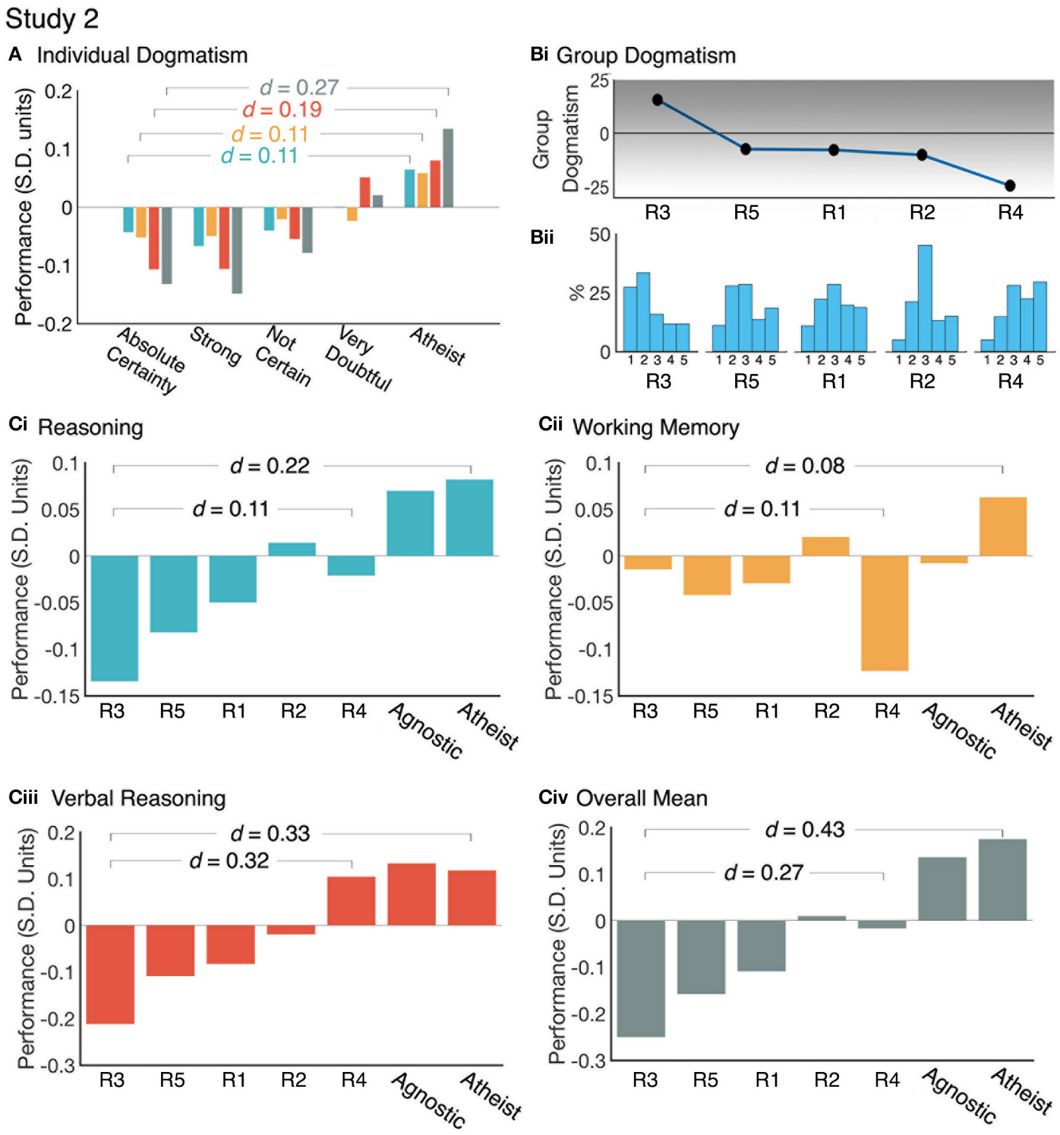
Examining the effect of religious dogmatism at the individual and religious group level. **(A)** Cognitive component performance by Individual self-rated religious dogmatism. Individuals with the highest religious dogmatism (Absolute Certainty) show significantly poorer performance scores than those with the lowest religious dogmatism (Atheist) in Verbal Reasoning and Overall Mean performance. **(Bi)** Religious groups were ranked by their Group dogmatism score calculated by the difference in proportions of the extreme belief responses. **(Bii)** Distributions individual dogmatism within each religious group (1 = Absolute Certainty, 2 = Strong, 3 = Not Certain, 4 = Very Doubtful, 5 = Atheist). **(Ci–Civ)** Cognitive performance varies across religious groups. Groups with larger proportions of individuals with strong religious beliefs show poorer performance, particularly in the Verbal Reasoning domain. Reasoning (blue), Working Memory (orange), Verbal Reasoning (red), and Overall Mean (gray) performance scores are in Standard Deviation units (Mean = 0, SD = 1).

**Figure 6:**
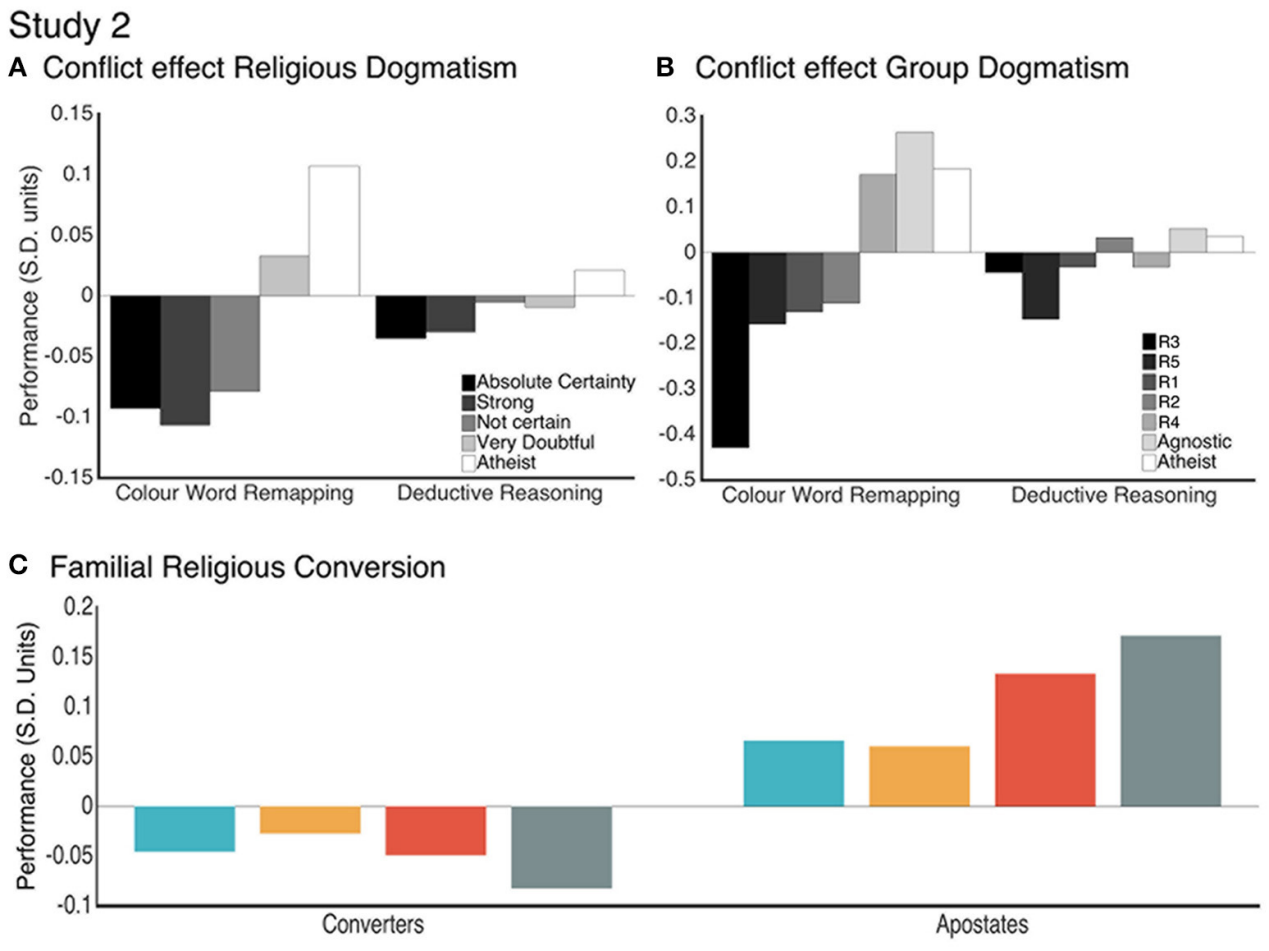
The interaction between task conflict and level of individual dogmatism **(A)** and group dogmatism **(B)**: The conflict task elicits pronounced group effects that is not observed in the non-conflict task. **(C)** Component scores compared between apostates (those from a religious family and are non-religious) and converters (those from a non-religious family and are religious). Reasoning (blue), Working Memory (orange), Verbal Reasoning (red), and Overall Mean (gray) performance scores are in Standard Deviation units (Mean = 0, SD = 1).

Study 2 included questions designed to examine the religiosity construct in more detail. One question that was framed as a Likert scale asked the participant to rate the strength of their religious belief (e.g., 1 = Absolute Certainty, 5 = Atheist), and this was taken as a proxy measure of religious dogmatism. Component scores from study 2 were binned according to the 5-point self-assessment. Cognitive components scores were each modeled as dependent variables in separate one-way ANOVAs with Individual Dogma as the between subject factor for both studies. Statistically significant main effects of Individual Dogma were found for all cognitive components (Supplementary Tables 16, 17). There was a clear pattern whereby cognitive performance increased as religious dogmatism decreased. Those with the greatest dogmatism were outperformed by those with the lowest dogmatism in Overall Mean (0.27 SDs) and Verbal Reasoning (0.19 SDs) scores (Figure 5A, Supplementary Table 12). Dogmatism showed a smaller relationship with Working Memory (0.11 SDs) and Reasoning (0.11 SDs) scores. Exemplifying the religious conflict effect, those with the greatest dogmatism were outperformed by those with the lowest dogmatism in tasks designed to manipulate conflict such as the CWR (0.20 SDs), Interlocking Polygons (0.24 SDs) and Grammatical Reasoning (0.23 SDs) (Figure 6A, Supplementary Table 18). Conversely, there were smaller differences in tasks that did not manipulate conflict; critically, this was the case for the Deductive Reasoning task (0.056 SDs).

### Examining the reasoning-religiosity relationship across religious groups

The questionnaire from study 2 also enabled religious individuals to be sub-divided according to religious groups (see Supplementary Table 1 for religious group sizes and age ranges. N.B. Those religious groups with *N* < 300 were dropped from our sample). We first assessed the effect of religious dogmatism on the religiosity-reasoning relationship at the group level. A “group dogmatism” score was calculated to rank the religious groups according to the difference in the proportion of low and high dogmatism individuals. Figure 5Bi reports how the distributions of individual dogmatism varied across the groups. The groups showed substantial differences in the average dogmatism scores of each religious group, with this effect spanning 0.87 SD units. This effect was reflected by correspondingly skewed cumulative distributions (Figure 5Bii). For example, Religious Group 1 (R1) had an approximately Gaussian distribution in terms of strength of belief. R4 had a distribution that was heavily skewed toward weak belief. R1 had a distribution that was heavily skewed toward strong belief.

Analysing the performance measures showed that the religious groups also differed significantly and that this pattern of differences reflected the observed variability in dogmatism. Specifically, the religious group with the highest mean dogmatism score was significantly outperformed by the religious group with the lowest mean dogmatism score in Verbal Reasoning (0.32 SDs), Overall Mean (0.23 SDs). Working Memory (0.11 SDs) and Reasoning (0.11 SDs) scores showed smaller effects (Figure 5C, Supplementary Table 12). Comparing the high dogmatism group to the Atheism group showed the most pronounced effects (Reasoning = 0.22 SDs, Working Memory = 0.08 SDs, Verbal Reasoning = 0.33 SDs, Overall Mean = 0.43 SDs).

Conflict detection effects were also compared across the religious groups. The high dogmatism group was outperformed by the low dogmatism group in tasks involving conflict detection such as the CWR (0.60 SDs), Grammatical Reasoning (0.29 SDs) and Interlocking Polygons (0.17 SDs) (Figure 6B, Supplementary Table 19). In contrast the high and low dogmatism groups did not differ in tasks that did not manipulate conflict, such as the Deductive Reasoning (0.01 SDs). The magnitude of these effects was greatest when comparing the high dogmatism group to the atheist group CWR (0.61 SDs), Grammatical Reasoning (0.36 SDs) and Interlocking Polygons (0.49 SDs). In contrast there was little difference between the highest dogmatism and the atheist groups for tasks without conflict, such as the Deductive Reasoning (0.08 SDs).

### Does religious conversion or apostasy relate to cognitive performance?

Finally, component scores were compared between those who have grown up in a religious family and are now non-religious (apostates) and those who have grown up in a non-religious family and are now religious (converters). The apostates showed greater component scores than the converters particularly within the Overall Mean (0.25 SDs) and Verbal Reasoning (0.18 SDs) and Reasoning (0.11 SDs) domains. A small difference was seen for Working Memory (0.08 SDs) (Figure 6C).

## Discussion

We tested multiple predictions regarding the cognitive mechanism underlying the relationship between religiosity and intelligence. The results accord well with the hypothesis that the religiosity effect reflects cognitive-behavioral biases that impair conflict detection (Gervais and Norenzayan, 2012; Pennycook et al., 2014), rather than general intelligence. These biases are most disadvantageous during tasks that are designed to introduce conflict between intuitive and logical answers.

Our analyses consistently confirmed that the non-religious groups have an advantage over religious groups in their overall mean performance of cognitive tasks. The scale of these effects was small but significant (Study 1 = 0.14 SDs; Study 2 = 0.27 SDs). This result accords with the ~2–4 IQ point differences previously reported between religious and atheist groups from large scale psychometric studies (Nyborg, 2009; Zuckerman et al., 2013). A *qualitative* comparison of the non-religious groups could lead to the interpretation that the atheist group outperforms the agnostic group both at the level of the latent variables and individual tasks. Despite this pattern being in accordance with the religious dogmatism's relationship to performance, a meaningful interpretation of this pattern is challenging due to the small effect size and the lack of consistency at the level of individual tasks across study 1 and 2.

Notably though, finer grained analyses of the data highlighted how comparing religious vs. non-religious groups in this manner underestimates the specificity and magnitude of the religiosity effect. Analysing religious dogmatism showed substantial differences across religions (0.87 SDs). This variability in dogmatism related significantly to the religiosity effect at the individual and the group level. The atheist group outperformed the most dogmatic group by 0.43 SDs in terms of overall mean score, which would be 6.45 IQ points. Previous studies demonstrated that individuals with low religious dogmatism score highly during analytic reasoning tasks (Gervais and Norenzayan, 2012; Shenhav et al., 2012) and on IQ (Lynn et al., 2009). Together, these consistent findings demonstrates failure to override incorrect intuitive responses correlates with religious dogmatism. Contrasting the relationships of religious dogmatism with performance between the levels of the individual differences and religious groups revealed an interesting pattern. The small scaled effects seen with Reasoning and Working Memory scores moved in opposing directions when elevated to the religious group level. Specifically, the Reasoning effect increased from a small to a medium scaled effect while the Working effect decreased from a small to a negligible effect. This pattern suggests that the relationship between religious dogmatism and the reasoning scores is more robust than with the Working Memory scores.

Our analyses extend the prior literature by demonstrating the highly robust and generalizable nature of the religiosity effect. The effect was reproducible across the two cohorts and evident after factoring out sociodemographic variables. Furthermore, the effect was not contingent on those variables, being robustly evident for all conditions of them. Critically, interactions between the religiosity effect and age or education level were statistically non-significant.

An interesting conclusion from this study is that the basis of the religiosity effect should be conceived of as a cognitive-behavioral bias, rather than impaired general intelligence. In examining the latent data structure, the religiosity effect showed a significantly scaled relationship with the reasoning components and little effect for the working memory component. This pattern could have reflected impaired reasoning ability in religious groups. However, not all tasks that loaded onto the reasoning latent variables showed a religiosity effect. The most striking example of this was the deductive reasoning task, a type of matrix reasoning task that we designed to have by far the most complex problems in our testing battery. This task requires that multiple rules, relating to different visual features (e.g., color and shape), be integrated as higher-order relational constructs. High dogmatism individuals and religious groups performed this task at a similar level to atheists.

Conversely, the CWR and grammatical reasoning tasks consistently showed some of the strongest religiosity effects. We intentionally designed these tasks to produce a conflict between alternative rule mappings. For example, the CWR is a challenging variant of the Stroop task (Stroop, 1935) that introduces a conflict between written to spoken word mappings and color naming. This variability in the magnitude of the religiosity effect across different reasoning-loaded tasks is most informative when compared with other sociodemographic variables. It is not the case that the matrix-reasoning task is unreliable or insensitive. Indeed, both age and computer game playing showed significantly scaled effects with both deductive reasoning and CWR task performance, suggesting that they relate to the ability that underlies this latent variable. Taken together, these results accord closely with the hypothesis that religious dogmatism correlates with a cognitive-behavioral tendency to forgo logical problem solving when an intuitive answer is available (Pennycook et al., 2014). In further support of this hypothesis we observed that religious apostates outperformed religious converts within the reasoning domains and that increased religious dogmatism relates to lower scores on the conflict, but not deductive reasoning, tasks at the individual and religious group levels. Comparing the highest dogma group to atheists showed a 0.61 SDs difference for the CWR task.

Our findings have significant implications for understanding the religiosity effects impact on higher cognition. From the dual-process perspective (Evans, 2008; Evans and Stanovich, 2013), failures in reasoning arise when fast intuitive processes are not overridden by slow logical processes. Individual differences in reasoning performance are therefore relative to an individuals cognitive capacity and style. Together, our findings suggest that the religiosity effect is not dependent on working memory laden logical processes but on the tendency to respond with an intuitive answer when intuitive and logical processes are in conflict.

Several limitations should be considered. Most notably, both of our cohorts were self-selecting populations of internet users which could have introduced sampling biases. However, the questionnaire data highlighted the wide variability and range of ages, education levels and countries of origin. This variability combined with the large cohort sizes allowed for these potential confounds to be factored out of the data prior to the analysis. Based on the robustness of the religiosity effect when accounting for other sociodemographic variables, it is highly unlikely that the religiosity effect has a basis in a confounding sociodemographic variable. Furthermore, when we took the largest and most heterogeneous religious group available, we observed that additionally factoring out race did not diminish the effect of religious dogmatism. Nonetheless, the non-random sampling method may have biased the distributions of dogmatism across religious groups; furthermore, religious groups likely vary in dogmatism dependent on region or sect. Consequently, it is important not to infer too strongly that the differences in religious dogmatism across groups extrapolate to the global population. Similarly, the small-to-medium group effects observed here mean that there is very substantial overlap across populations in terms of cognitive performances. It is therefore inappropriate to generalize these effects to specific individuals.

Finally, a limitation for any observational and cross-sectional study is that cause and effect cannot be directly inferred from correlational analyses. Future work may adopt interventional approaches to examine causal relationships. Indeed, if the religiosity effect is based on learnt cognitive-behavioral biases, then this holds some hope. Humans are exceedingly capable of resolving maladaptive cognition via training therapies. In contrast, the question of whether it is possible to train core abilities remains highly controversial (Owen et al., 2010; Simons et al., 2016). An interesting future study could determine whether cognitive training can ameliorate the religiosity effect by enabling individuals to apply their latent reasoning abilities, even when there appears to be intuitive answers. A previous study by Gervais and Norenzayan (2012) provides preliminary support for this view. They examined the causal relationship between religious dogmatism and reasoning by exposing individuals to exercises in analytical thinking. In the period post exercise, reductions in religious dogmatism were evident. A timely question, is whether repeat exercise might lead to lasting benefits in conflict detection, with consequently generalized improvements in cognitive task performance.

In conclusion, religiosity is associated with poorer reasoning performance during tasks that involve cognitive conflict. These effects may reflect learnt cognitive-behavioral biases toward intuitive decision making, rather than underlying abilities to understand complex logical rules or to maintain information in working memory. The effects are consistent in two large cohorts and robust across sociodemographic variables. Future work may focus on deconstructing the religiosity and dogmatism constructs in greater detail (Evans, 2001; Whitehouse, 2002, 2004; Friedman and Rholes, 2007), determining how the impact of these on real-world achievement is mediated by cognitive behavior, and testing whether cognitive training may counter biases of the religious mind toward intuitive decision-making.

## Author contributions

AH programmed the servers, adapted the cognitive tasks for the Internet and facilitated the data acquisition for both studies. AH curated the hypotheses of interest. RD preprocessed and analyzed the study data, and produced all figures and tables. Both RD and AH drafted the manuscript and approved the final version of the manuscript prior to submission.

### Conflict of interest statement

The authors declare that the research was conducted in the absence of any commercial or financial relationships that could be construed as a potential conflict of interest.

## References

[B1] Argyle M. (1958). Religious Behaviour. London: Routledge.

[B2] Asp E. Ramchandran K. Tranel D. (2012). Authoritarianism, religiousfundamentalism, and the human prefrontalcortex. Neuropsychology 26, 414–421. 10.1037/a0028526 22612576 PMC3389201

[B3] Baddeley A. D. (1968). A three-minute reasoning test based on grammatical transformation. Psychometr. Sci. 10, 341–342. 10.3758/BF03331551

[B4] Carlucci L. Tommasi M. Balsamo M. Furnham A. Saggino A. (2015). Religious fundamentalism and psychological well-being: an Italian study. J. Psychol. Theol. 43, 22–33.

[B5] Carlucci L. Tommasi M. Saggino A. (2011). Socio-demographic and five factor model variables as predictors of religious fundamentalism: an italian study. Arch. Psychol. Relig. 33, 253–268. 10.1163/157361211X576609

[B6] Colzato L. S. Van Den Wildenberg W. P. Hommel B. (2008). Losing the big picture: how religion can control visual attention. PLoS ONE 3:e3679. 10.1371/journal.pone.0003679 19002253 PMC2577734

[B7] Dawkins R. (2008). The God Delusion, 1st Edn., Mariner Books. Boston: Houghton Mifflin Co.

[B8] Dennett D. C. (2006). Breaking the Spell: Religion as a Natural Phenomenon. New York, NY: Viking.

[B9] Ecklund E. H. Johnson D. R. Scheitle C. P. Matthews K. R. W. Lewis S. W. (2016). Religion among scientists in international context: a new study of scientists in eight regions. Socius 2, 1–9. 10.1177/2378023116664353

[B10] Evans E. M. (2001). Cognitive and contextual factors in the emergence of diverse belief systems: creation versus evolution. Cogn. Psychol. 42, 217–266. 10.1006/cogp.2001.0749 11305883

[B11] Evans J. S. (2008). Dual-processing accounts of reasoning, judgment, and social cognition. Annu. Rev. Psychol. 59, 255–278. 10.1146/annurev.psych.59.103006.093629 18154502

[B12] Evans J. S. Stanovich K. E. (2013). Dual-process theories of higher cognition: advancing the debate. Perspect. Psychol. Sci. 8, 223–241. 10.1177/1745691612460685 26172965

[B13] Friedman M. Rholes W. S. (2007). Successfully challenging fundamentalist beliefs results in increased death awareness. J. Exp. Soc. Psychol. 43, 794–801. 10.1016/j.jesp.2006.07.008

[B14] Galen L. W. Wolfe M. B. Deleeuw J. Wyngarden N. (2009). Religious fundamentalism as schema: influences on memory for religious information. J. Appl. Soc. Psychol. 39, 1163–1190. 10.1111/j.1559-1816.2009.00476.x

[B15] Gervais W. M. Norenzayan A. (2012). Analytic thinking promotes religious disbelief. Science 336, 493–496. 10.1126/science.1215647 22539725

[B16] Hampshire A. Highfield R. R. Parkin B. L. Owen A. M. (2012a). Fractionating human intelligence. Neuron 76, 1225–1237. 10.1016/j.neuron.2012.06.022 23259956

[B17] Hampshire A. Parkin B. L. Cusack R. Espejo D. F. Allanson J. Kamau E.. (2012b). Assessing residual reasoning ability in overtly non-communicative patients using fMRI. Neuroimage Clin. 2, 174–183. 10.1016/j.nicl.2012.11.008 24179769 PMC3777757

[B18] Harris S. (2004). The End of Faith: Religion, Terror, and the Future of Reason, 1st Edn., New York, NY: W.W. Norton & Co.

[B19] Hitchens C. (2007). God Is Not Great: How Religion Poisons Everything, 1st Edn. New York, NY: Twelve.

[B20] Howells T. H. (1928). A Comparative Study of Those Who Accept As against Those Who Reject Religious Authority. University of Iowa Studies of Character.

[B21] Larson E. J. Witham L. (1998). Leading Scientists still reject God. Nature 394:313 10.1038/284789690462

[B22] Lynn R. Harvey J. Nyborg H. (2009). Average intelligence predicts atheism rates across 137 nations. Intelligence 37, 11–15. 10.1016/j.intell.2008.03.004

[B23] Morgan J. (2014). Religion and dual-process cognition: a continuum of styles or distinct types? Relig. Brain Behav. 6, 112–129. 10.1080/2153599X.2014.966315

[B24] Norenzayan A. Gervais W. M. (2013). The origins of religious disbelief. Trends Cogn. Sci. 17, 20–25. 10.1016/j.tics.2012.11.006 23246230

[B25] Nyborg H. (2009). The intelligence–religiosity nexus: a representative study of white adolescent Americans. Intelligence 37, 81–93. 10.1016/j.intell.2008.08.003

[B26] Oviedo L. (2015). Religious cognition as a dual-process: developing the model. Method Theor. Study Relig. 27, 31–58. 10.1163/15700682-12341288

[B27] Owen A. M. Hampshire A. Grahn J. A. Stenton R. Dajani S. Burns A. S.. (2010). Putting brain training to the test. Nature 465, 775–778. 10.1038/nature09042 20407435 PMC2884087

[B28] Pargament K. L. Smith B. W. Koenig H. G. Perez L. (1998). Patterns of positive and negative religious coping with major life stressors. J. Sci. Study f Relig. 37, 710–724. 10.2307/1388152

[B29] Pennycook G. Cheyne J. A. Barr N. Koehler D. J. Fugelsang J. A. (2014). Cognitive style and religiosity: the role of conflict detection. Mem. Cognit. 42, 1–10. 10.3758/s13421-013-0340-7 23784742

[B30] Pennycook G. Cheyne J. A. Koehler D. J. Fugelsang J. A. (2013). Belief bias during reasoning among religious believers and skeptics. Psychon. Bull. Rev. 20, 806–811. 10.3758/s13423-013-0394-3 23397237

[B31] Razmyar S. Reeve C. L. (2013). Individual differences in religiosity as a function of cognitive ability and cognitive style. Intelligence 41, 667–673. 10.1016/j.intell.2013.09.003

[B32] Saroglou V. (2002). Religion and the five factors of personality: a meta-analytic review. Pers. Individ. Dif. 32, 15–25. 10.1016/S0191-8869(00)00233-6

[B33] Shenhav A. Rand D. G. Greene J. D. (2012). Divine intuition: cognitive style influences belief in God. J. Exp. Psychol. Gen. 141, 423–428. 10.1037/a0025391 21928924

[B34] Simons D. J. Boot W. R. Charness N. Gathercole S. E. Chabris C. F. Hambrick D. Z.. (2016). Do “brain-training” programs work? Psychol. Sci. Public Interest 17, 103–186. 10.1177/1529100616661983 27697851

[B35] Sinclair R. D. (1928). A Comparative Study of Those Who Report the Experience of the Divine Prescence with Those Who do Not. University of Iowa Studies of Character.

[B36] Stanovich K. E. West R. F. (1998). Individual differences in rational thought. J. Exp. Psychol. 127, 161–188. 10.1037/0096-3445.127.2.161

[B37] Stirrat M. Cornwell R. E. (2013). Eminent scientists reject the supernatural: a survey of the Fellows of the Royal Society. Evolution 6:33 10.1186/1936-6434-6-33

[B38] Stroop J. R. (1935). Studies of interference in serial verbal reactions. J. Exp. Psychol. 18:643 10.1037/h0054651

[B39] Verhage F. (1964). Intelligence and religious persuasion. Nederlands tijdschift voor de Psychologie en haar Grensgebieden 19, 247–254. 14168788

[B40] Whitehouse H. (2002). Modes of Religiosity: towards a cognitive explanation of the sociopolitical dynamics of religion. Method Theory Study Relig. 14, 293–315. 10.1163/157006802320909738

[B41] Whitehouse H. (2004). Modes of Religiosity: A Cognitive Theory of Religious Transmission. Leiden: BRILL.

[B42] Zhong W. Cristofori I. Bulbulia J. Krueger F. Grafman J. (2017). Biological and cognitive underpinnings of religiousfundamentalism. Neuropsychology 100, 18–25. 10.1016/j.neuropsychologia.2017.04.009 28392301 PMC5500821

[B43] Zuckerman M. Silberman J. Hall J. A. (2013). The relation between intelligence and religiosity: a meta-analysis and some proposed explanations. Pers. Soc. Psychol. Rev. 17, 325–354. 10.1177/1088868313497266 23921675

