# Supplementary material for: The Negative Relationship between Reasoning and Religiosity Is Underpinned by a Bias for Intuitive Responses Specifically When Intuition and Logic Are in Conflict
Source: Front Psychol. 2017 Dec 19;8:2191. doi: 10.3389/fpsyg.2017.02191 (PMC5742220; doi:10.3389/fpsyg.2017.02191)
Supplement: Supplementary file 1 [file Table1.docx]

***­Supplementary Materials***

The negative relationship between reasoning and religiosity is underpinned by a bias for intuitive responses specifically when intuition and logic are in conflict

Richard E. Daws^1^ & Adam Hampshire^1*^

*Corresponding Author: [a.hampshire@imperial.ac.uk](mailto:a.hampshire@imperial.ac.uk)

^1^The Computational, Cognitive & Clinical Neuroimaging Laboratory (C^3^NL), Imperial College London, London, UK.


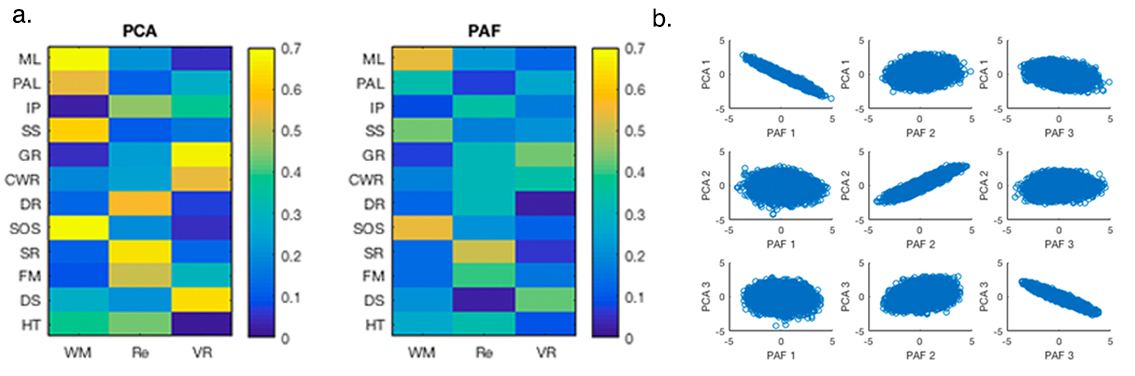


Supplemental Figure 1. Comparison of Principal Component Analysis (PCA) & Principal Axis Factoring (PAF) as dimensionality reducing techniques. In both cases the varimax algorithm was applied to significant components as defined by the Kaiser convention (EVs≥1). a) Comparison of the rotated loading matrices (abs. values). b) Component scores plotted against Factor scores.

Supplementary table 1. Number of participants within each group, mean age and its Standard Deviation (St.D). The religious group in Study 2 is composed of the religious subgroups.

|  |  | Age | | |
| --- | --- | --- | --- | --- |
|  | N | Mean | St.D | |
| ***Study 1*** |  |  |  | |
| Religious | 12,576 | 31.38 | 12.02 | |
| Agnostic | 14,018 | 30.12 | 10.99 | |
| Atheist | 18,186 | 29.98 | 11.26 | |
| ***Study 2*** |  |  |  | |
| Religious | 10,876 | 34.02 | 14.26 | |
| Agnostic | 2,612 | 30.44 | 12.31 | |
| Atheist | 4,967 | 29.73 | 11.86 | |
| ***Study 2 – Religious subgroups*** | | | |  |
| R1 | 9,230 | 34.71 | 14.43 | |
| R2 | 476 | 28.81 | 10.82 | |
| R3 | 410 | 27.11 | 9.20 | |
| R4 | 393 | 36.93 | 16.90 | |
| R5 | 367 | 28.06 | 9.93 | |

Supplementary table 2. Education breakdown: Percentages of subjects at each previously attained level of education.

|  | Some High School | High School Graduate | Some College | College Graduate | Higher |  |  |
| --- | --- | --- | --- | --- | --- | --- | --- |
| ***Study 1*** | |  |  |  |  |  |  |
| Religious | 8.36 | 8.78 | 24.6 | 31.55 | 26.71 |  |  |
| Agnostic | 7.31 | 9.34 | 27.64 | 31.15 | 24.55 |  |  |
| Atheist | 8.45 | 9.42 | 27.16 | 28.46 | 26.51 |  |  |
|  | |  |  |  |  |  |  |
|  | Some School | School Graduate | Some College | College Graduate | Some University | University Graduate | Higher |
| ***Study 2*** |  |  |  |  |  |  |  |
| Religious | 5.86 | 8.42 | 15.82 | 11.92 | 18.88 | 26.19 | 12.91 |
| Agnostic | 5.74 | 8.38 | 16.88 | 10.91 | 17.73 | 25.69 | 14.66 |
| Atheist | 4.93 | 7.88 | 13.74 | 12.92 | 15.95 | 30.96 | 13.63 |
| ***Study 2 - Religious subgroups*** | | |  |  |  |  |  |
| R1 | 4.56 | 8.14 | 13.88 | 13.1 | 16.13 | 31.02 | 13.17 |
| R3 | 8.78 | 8.78 | 11.95 | 9.76 | 17.56 | 30.73 | 12.44 |
| R4 | 6.62 | 5.85 | 11.2 | 9.92 | 10.94 | 27.48 | 27.99 |
| R5 | 3.54 | 4.09 | 12.81 | 16.35 | 8.72 | 37.87 | 16.62 |
| R2 | 8.4 | 6.72 | 15.34 | 11.97 | 20.8 | 27.52 | 9.24 |

Supplementary table 3. Country of Origin breakdown: Percentages of subject within each group and global region.

|  | UK | USA | Africa | | W. Europe | | E. Europe | | Aus / NZ | | S. America | | Asia | | Middle East | | Other | |  |
| --- | --- | --- | --- | --- | --- | --- | --- | --- | --- | --- | --- | --- | --- | --- | --- | --- | --- | --- | --- |
| ***Study 1*** |  |  |  | |  | |  | |  | |  | |  | |  | |  | |  |
| Religious | 15.46 | 39.73 | 2.07 | | 11.28 | | 8.15 | | 9.65 | | 2.93 | | 7.98 | | 1.53 | | 1.22 | |  |
| Agnostic | 19.63 | 41.7 | 1.04 | | 12.27 | | 6.66 | | 10.3 | | 2.21 | | 4.52 | | 0.83 | | 0.83 | |  |
| Atheist | 27.27 | 31.92 | 1.25 | | 14.23 | | 6.22 | | 12.05 | | 1.94 | | 3.64 | | 0.73 | | 0.75 | |  |
|  | UK | USA | Africa | | W. Europe | | E. Europe | | Aus / NZ | | S. America | | Asia | | Middle East | |  | |  |
| ***Study 2*** |  |  |  | |  | |  | |  | |  | |  | |  | |  | |  |
| Religious | 8.04 | 41.95 | 0.79 | | 18.1 | | 11.35 | | 2.39 | | 7.97 | | 7.79 | | 1.63 | |  | |  |
| Agnostic | 10.68 | 50.88 | 0.38 | | 17.42 | | 6.55 | | 3.68 | | 5.51 | | 4.02 | | 0.88 | |  | |  |
| Atheist | 13.27 | 41.51 | 0.24 | | 22.23 | | 9 | | 3.95 | | 3.74 | | 5.34 | | 0.72 | |  | |  |
| ***Study 2 - Religious subgroups*** | | | |  | |  | |  | |  | |  | |  | |  | |  | |
| R1 | 8.09 | 43.35 | 0.47 | | 20.2 | | 12.76 | | 2.44 | | 8.95 | | 3.46 | | 0.29 | |  | |  |
| R3 | 9.02 | 16.83 | 6.83 | | 9.51 | | 4.88 | | 1.46 | | 1.22 | | 21.46 | | 28.78 | |  | |  |
| R4 | 9.41 | 67.94 | 0.76 | | 3.56 | | 2.8 | | 2.04 | | 3.31 | | 3.56 | | 6.62 | |  | |  |
| R5 | 6.54 | 15.26 | 2.18 | | 1.36 | | 1.63 | | 0.82 | | 1.36 | | 69.48 | | 1.36 | |  | |  |
| R2 | 6.09 | 35.5 | 0.84 | | 9.87 | | 3.99 | | 3.78 | | 3.78 | | 35.92 | | 0.21 | |  | |  |

Supplementary table 4. Main effect of Religious Group on Age (years).

| **Main Effect of Religious Group on Age** | df | F | p |
| --- | --- | --- | --- |
| ***Study 1*** |  |  |  |
| Religious Group | 2 | 49.040 | <0.0001 |
| Error | 44362 |  |  |
| Total | 44364 |  |  |
| ***Study 2*** |  |  |  |
| Religious Group | 2 | 206.532 | <0.0001 |
| Error | 18417 |  |  |
| Total | 18419 |  |  |
| ***Study 2 – Religious subgroups*** |  |  |  |
| Religious Group | 6 | 120.108 | <0.0001 |
| Error | 18413 |  |  |
| Total | 18419 |  |  |

Supplementary table 5. Religious group pairwise comparisons of group mean age using cohen’s d t-statistics and group age distributions with ks-statistics.

| **Age Comparisons** | | | ***d* (abs.)** | t | p | ks | p |
| --- | --- | --- | --- | --- | --- | --- | --- |
| ***Study 1*** | | | | | | | |
| Religious | vs. | Atheist | 0.12 | 10.364 | p<0.001 | 0.051 | p<0.001 |
| Religious | vs. | Agnostic | 0.10 | 8.895 | p<0.001 | 0.052 | p<0.001 |
| Atheist | vs. | Agnostic | 0.01 | -1.092 | 0.275 | 0.023 | p<0.001 |
| ***Study 2*** | | | | | | | |
| Religious | vs. | Atheist | 0.33 | 18.494 | p<0.001 | 0.136 | p<0.001 |
| Religious | vs. | Agnostic | 0.27 | 11.811 | p<0.001 | 0.114 | p<0.001 |
| Atheist | vs. | Agnostic | 0.05 | -2.465 | 0.014 | 0.029 | 0.122 |
| ***Study 2 - subgroups*** | | | | | | | |
| R1 | vs. | Atheism | 0.37 | 20.842 | p<0.001 | 0.16 | p<0.001 |
| R1 | vs. | Agnostic | 0.31 | 13.767 | p<0.001 | 0.138 | p<0.001 |
| R1 | vs. | R2 | 0.46 | 8.796 | p<0.001 | 0.191 | p<0.001 |
| R1 | vs. | R3 | 0.62 | 10.554 | p<0.001 | 0.241 | p<0.001 |
| R1 | vs. | R4 | 0.14 | -2.943 | 0.003 | 0.085 | 0.009 |
| R1 | vs. | R5 | 0.53 | 8.723 | p<0.001 | 0.215 | p<0.001 |
| Atheism | vs. | Agnostic | 0.05 | -2.465 | 0.014 | 0.029 | 0.122 |
| Atheism | vs. | R2 | 0.08 | 1.628 | 0.104 | 0.037 | 0.581 |
| Atheism | vs. | R3 | 0.24 | 4.345 | p<0.001 | 0.085 | 0.008 |
| Atheism | vs. | R4 | 0.49 | -11.128 | p<0.001 | 0.196 | p<0.001 |
| Atheism | vs. | R5 | 0.15 | 2.611 | 0.009 | 0.067 | 0.089 |
| Agnostic | vs. | R2 | 0.14 | 2.714 | 0.007 | 0.058 | 0.127 |
| Agnostic | vs. | R3 | 0.30 | 5.242 | p<0.001 | 0.106 | p<0.001 |
| Agnostic | vs. | R4 | 0.43 | -9.186 | p<0.001 | 0.176 | p<0.001 |
| Agnostic | vs. | R5 | 0.21 | 3.535 | p<0.001 | 0.082 | 0.025 |
| R2 | vs. | R3 | 0.16 | 2.479 | 0.013 | 0.076 | 0.153 |
| R2 | vs. | R4 | 0.57 | -8.55 | p<0.001 | 0.215 | p<0.001 |
| R2 | vs. | R5 | 0.07 | 1.02 | 0.308 | 0.056 | 0.534 |
| R3 | vs. | R4 | 0.72 | -10.25 | p<0.001 | 0.267 | p<0.001 |
| R3 | vs. | R5 | 0.09 | -1.378 | 0.169 | 0.051 | 0.685 |
| R4 | vs. | R5 | 0.63 | 8.706 | p<0.001 | 0.255 | p<0.001 |

Supplementary table 6. χ^2^ test of independence statistics for Level of Education, Country of Origin (summed across global region) & Ethnicity.

|  | df | χ^2^ | p |
| --- | --- | --- | --- |
| ***Level of Education*** | |  |  |
| Study 1 | 8 | 90.65 | <0.05 |
| Study 2 | 12 | 95.7 | <0.05 |
| Study 2 – subgroups | 36 | 253.17 | <0.05 |
| ***Country of Origin*** | |  |  |
| Study 1 | 18 | 1338.9 | <0.05 |
| Study 2 | 18 | 468.42 | <0.05 |
| Study 2 – subgroups | 54 | 6979.6 | <0.05 |
| ***Ethnicity*** |  |  |  |
| Study 1 | 10 | 785.32 | <0.05 |
| Study 2 | 12 | 383.59 | <0.05 |
| Study 2 – subgroups | 36 | 4880.9 | <0.05 |

Supplementary table 7. Task cross-correlation matrices for both Study’s (Pearson R2) (ML=Monkey Ladder, PAL = Paired Associate Leaning, IP=Interlocking Polygons, SS=Spatial Span, GR=Grammatical Reasoning, CWR=Colour Word Remapping, DR=Deductive Reasoning, SOS=Self Ordered Search, SR=Spatial Reasoning, FM=Feature Match, DS=Digit Span, HTT=Hampshire Tree Task, MF=Moving Flanker, AR=Analogical Reasoning, SC=Spatial Chunking).

| **Study 1** | ML | PAL | IP | SS | GR | CWR | DR | SOS | SR | FM | DS |  |
| --- | --- | --- | --- | --- | --- | --- | --- | --- | --- | --- | --- | --- |
| PAL | 0.263 |  |  |  |  |  |  |  |  |  |  |  |
| IP | 0.168 | 0.124 |  |  |  |  |  |  |  |  |  |  |
| SS | 0.324 | 0.253 | 0.182 |  |  |  |  |  |  |  |  |  |
| GR | 0.215 | 0.170 | 0.192 | 0.203 |  |  |  |  |  |  |  |  |
| CWR | 0.252 | 0.193 | 0.213 | 0.267 | 0.325 |  |  |  |  |  |  |  |
| DR | 0.191 | 0.122 | 0.155 | 0.149 | 0.156 | 0.146 |  |  |  |  |  |  |
| SOS | 0.413 | 0.250 | 0.184 | 0.339 | 0.184 | 0.260 | 0.184 |  |  |  |  |  |
| SR | 0.221 | 0.141 | 0.233 | 0.196 | 0.219 | 0.229 | 0.236 | 0.230 |  |  |  |  |
| FM | 0.238 | 0.164 | 0.240 | 0.205 | 0.227 | 0.253 | 0.198 | 0.241 | 0.270 |  |  |  |
| DS | 0.174 | 0.206 | 0.103 | 0.175 | 0.217 | 0.181 | 0.060 | 0.171 | 0.066 | 0.104 |  |  |
| HTT | 0.278 | 0.184 | 0.180 | 0.261 | 0.200 | 0.256 | 0.189 | 0.267 | 0.260 | 0.223 | 0.079 |  |
|  |  |  |  |  |  |  |  |  |  |  |  |  |
| **Study 2** | ML | IP | GR | CWR | DR | SOS | SR | FM | DS | HTT | SC | MF |
| IP | 0.136 |  |  |  |  |  |  |  |  |  |  |  |
| GR | 0.206 | 0.187 |  |  |  |  |  |  |  |  |  |  |
| CWR | 0.183 | 0.168 | 0.282 |  |  |  |  |  |  |  |  |  |
| DR | 0.144 | 0.139 | 0.105 | 0.113 |  |  |  |  |  |  |  |  |
| SOS | 0.245 | 0.134 | 0.206 | 0.164 | 0.096 |  |  |  |  |  |  |  |
| SR | 0.172 | 0.210 | 0.183 | 0.172 | 0.212 | 0.142 |  |  |  |  |  |  |
| FM | 0.215 | 0.224 | 0.205 | 0.201 | 0.173 | 0.144 | 0.243 |  |  |  |  |  |
| DS | 0.168 | 0.084 | 0.166 | 0.210 | 0.037 | 0.144 | 0.030 | 0.096 |  |  |  |  |
| HTT | 0.237 | 0.168 | 0.237 | 0.173 | 0.174 | 0.190 | 0.247 | 0.183 | 0.073 |  |  |  |
| SC | 0.333 | 0.142 | 0.193 | 0.105 | 0.121 | 0.230 | 0.169 | 0.177 | 0.136 | 0.213 |  |  |
| MF | 0.232 | 0.173 | 0.256 | 0.226 | 0.120 | 0.202 | 0.167 | 0.243 | 0.145 | 0.192 | 0.198 |  |
| AR | 0.183 | 0.204 | 0.244 | 0.201 | 0.149 | 0.181 | 0.248 | 0.182 | 0.102 | 0.214 | 0.201 | 0.206 |

Supplementary table 8. Unrotated and rotated principal component matrices from Study’s 1 & 2.

| **Study 1** | | | | | | | | | |  | | | | |  | | | | | | | | | | | | | | | | |
| --- | --- | --- | --- | --- | --- | --- | --- | --- | --- | --- | --- | --- | --- | --- | --- | --- | --- | --- | --- | --- | --- | --- | --- | --- | --- | --- | --- | --- | --- | --- | --- |
| Unrotated | WM | | Re | | VR | |  | | | | Rotated | | | | | WM | | | | Re | | | | VR | | | |  |  |  |  |
| Monkey Ladder | 0.621 | | 0.190 | | -0.329 | |  | | | | Monkey Ladder | | | | | 0.693 | | | | 0.208 | | | | 0.075 | | | |  |  |  |  |
| Paired Associates Learning | 0.478 | | 0.402 | | -0.125 | |  | | | | Paired Associates Learning | | | | | 0.578 | | | | -0.033 | | | | 0.265 | | | |  |  |  |  |
| Interlocking Polygons | 0.456 | | -0.311 | | 0.266 | |  | | | | Interlocking Polygons | | | | | 0.003 | | | | 0.542 | | | | 0.284 | | | |  |  |  |  |
| Spatial Span | 0.583 | | 0.232 | | -0.219 | |  | | | | Spatial Span | | | | | 0.624 | | | | 0.159 | | | | 0.165 | | | |  |  |  |  |
| Grammatical Reasoning | 0.522 | | 0.018 | | 0.528 | |  | | | | Grammatical Reasoning | | | | | 0.048 | | | | 0.342 | | | | 0.658 | | | |  |  |  |  |
| Colour Word Remapping | 0.582 | | 0.021 | | 0.303 | |  | | | | Colour Word Remapping | | | | | 0.221 | | | | 0.361 | | | | 0.502 | | | |  |  |  |  |
| Deductive Reasoning | 0.417 | | -0.383 | | -0.143 | |  | | | | Deductive Reasoning | | | | | 0.182 | | | | 0.547 | | | | -0.093 | | | |  |  |  |  |
| Self Ordered Search | 0.619 | | 0.172 | | -0.350 | |  | | | | Self Ordered Search | | | | | 0.697 | | | | 0.219 | | | | 0.050 | | | |  |  |  |  |
| Spatial Rotations | 0.526 | | -0.433 | | 0.006 | |  | | | | Spatial Rotations | | | | | 0.145 | | | | 0.663 | | | | 0.056 | | | |  |  |  |  |
| Feature Match | 0.539 | | -0.290 | | 0.112 | |  | | | | Feature Match | | | | | 0.158 | | | | 0.566 | | | | 0.204 | | | |  |  |  |  |
| Digit Span | 0.360 | | 0.549 | | 0.429 | |  | | | | Digit Span | | | | | 0.246 | | | | -0.182 | | | | 0.722 | | | |  |  |  |  |
| Hampshire Tree | 0.549 | | -0.157 | | -0.224 | |  | | | | Hampshire Tree | | | | | 0.422 | | | | 0.444 | | | | -0.011 | | | |  |  |  |  |
| **Study 2** | | | | | | | | |  | | | | |  | | | | |  | | | |  | | | |  | | | |  |
| Unrotated | | Re | | WM | | VR | |  | | | | Rotated | | | | | Re | | | | WM | | | | VR | | | |  |  |  |
| Monkey Ladder | | 0.554 | | 0.212 | | -0.412 | |  | | | | | Monkey Ladder | | | | | 0.138 | | | | 0.693 | | | | 0.150 | | | |  |  |
| Interlocking Polygons | | 0.454 | | -0.256 | | 0.257 | |  | | | | | Interlocking Polygons | | | | | 0.509 | | | | -0.029 | | | | 0.279 | | | |  |  |
| Grammatical Reasoning | | 0.559 | | 0.162 | | 0.249 | |  | | | | | Grammatical Reasoning | | | | | 0.250 | | | | 0.179 | | | | 0.553 | | | |  |  |
| Colour Word Remapping | | 0.499 | | 0.199 | | 0.493 | |  | | | | | Colour Word Remapping | | | | | 0.210 | | | | -0.026 | | | | 0.697 | | | |  |  |
| Deductive Reasoning | | 0.371 | | -0.449 | | -0.087 | |  | | | | | Deductive Reasoning | | | | | 0.570 | | | | 0.117 | | | | -0.092 | | | |  |  |
| Self Ordered Search | | 0.479 | | 0.264 | | -0.272 | |  | | | | | Self Ordered Search | | | | | 0.066 | | | | 0.564 | | | | 0.226 | | | |  |  |
| Spatial Rotations | | 0.504 | | -0.465 | | 0.017 | |  | | | | | Spatial Rotations | | | | | 0.676 | | | | 0.107 | | | | 0.040 | | | |  |  |
| Feature Match | | 0.521 | | -0.198 | | 0.139 | |  | | | | | Feature Match | | | | | 0.494 | | | | 0.117 | | | | 0.270 | | | |  |  |
| Digit Span | | 0.328 | | 0.591 | | 0.224 | |  | | | | | Digit Span | | | | | -0.229 | | | | 0.218 | | | | 0.638 | | | |  |  |
| Hampshire Tree | | 0.528 | | -0.172 | | -0.185 | |  | | | | | Hampshire Tree | | | | | 0.443 | | | | 0.374 | | | | 0.078 | | | |  |  |
| Spatial Chunking | | 0.509 | | 0.158 | | -0.534 | |  | | | | | Spatial Chunking | | | | | 0.138 | | | | 0.742 | | | | 0.017 | | | |  |  |
| Moving Flanker | | 0.538 | | 0.140 | | 0.111 | |  | | | | | Moving Flanker | | | | | 0.240 | | | | 0.264 | | | | 0.441 | | | |  |  |
| Analogical Reasoning | | 0.528 | | -0.137 | | 0.085 | |  | | | | | Analogical Reasoning | | | | | 0.444 | | | | 0.183 | | | | 0.271 | | | |  |  |

Supplementary table 9. Analysis of Variance examining the effect of Religious group on components of cognition scores.

| ***Main effects of Religious Group on components of cognition*** | | | | | | | | | |
| --- | --- | --- | --- | --- | --- | --- | --- | --- | --- |
| ***Study 1*** | | | | ***Study 2*** | | | ***Study 2 -  Religious sub groups*** | | |
| ***Reasoning*** | df | F | p | df | F | p | df | F | p |
| Religious Group | 2 | 132.477 | <0.0001 | 2 | 86.277 | <0.0001 | 6 | 30.960 | <0.0001 |
| Error | 44777 |  |  | 18452 |  |  | 18448 |  |  |
| Total | 44779 |  |  | 18454 |  |  | 18454 |  |  |
| ***Working Memory*** |  |  |  |  |  |  |  |  |  |
| Religious Group | 2 | 4.236 | 0.014 | 2 | 16.178 | <0.0001 | 6 | 6.249 | <0.0001 |
| Error | 44777 |  |  | 18452 |  |  | 18448 |  |  |
| Total | 44779 |  |  | 18454 |  |  | 18454 |  |  |
| ***Verbal Reasoning*** |  |  |  |  |  |  |  |  |  |
| Religious Group | 2 | 92.476 | <0.0001 | 2 | 142.464 | <0.0001 | 6 | 53.448 | <0.0001 |
| Error | 44777 |  |  | 18452 |  |  | 18448 |  |  |
| Total | 44779 |  |  | 18454 |  |  | 18454 |  |  |
| ***Overall Mean*** |  |  |  |  |  |  |  |  |  |
| Religious Group | 2 | 167.106 | <0.0001 | 2 | 197.326 | <0.0001 | 6 | 69.686 | <0.0001 |
| Error | 44777 |  |  | 18452 |  |  | 18448 |  |  |
| Total | 44779 |  |  | 18454 |  |  | 18454 |  |  |

Supplementary table 10. Pairwise comparisons between the Religious Groups component scores. T-tests statistics (t) were calculated to assess whether means differed between groups & Kolmogorov-Sminov (ks) statistics were calculated to assess whether the two samples came from independent distributions.

| ***Study 1*** |  |  |  |  | ***Study 2*** | | | | |  | | |  | |  |
| --- | --- | --- | --- | --- | --- | --- | --- | --- | --- | --- | --- | --- | --- | --- | --- |
|  | t | p | ks | p | t | | p | | ks | | | p | |  |  |
| ***Reasoning*** |  |  |  |  |  | |  | |  | | |  | |  |  |
| Atheist vs. Agnostic | 6.132 | <0.0001 | 0.030 | <0.0001 | -8.480 | | <0.0001 | | 0.082 | | | <0.0001 | |  |  |
| Atheist vs. Religious | 16.244 | <0.0001 | 0.074 | <0.0001 | -11.828 | | <0.0001 | | 0.091 | | | <0.0001 | |  |  |
| Agnostic vs. Religious | 9.747 | <0.0001 | 0.049 | <0.0001 | -0.762 | | 0.446 | | 0.024 | | | 0.295 | |  |  |
| ***Working Memory*** |  |  |  |  |  |  | |  | | |  |  |  |  |  |
| Atheist vs. Agnostic | 2.907 | 0.003 | 0.021 | 0.001 | -1.065 | | 0.287 | | 0.016 | | | 0.640 | |  |  |
| Atheist vs. Religious | 0.977 | 0.328 | 0.010 | 0.386 | -5.635 | | <0.0001 | | 0.038 | | | <0.0001 | |  |  |
| Agnostic vs. Religious | -1.723 | 0.084 | 0.017 | 0.033 | -3.126 | | 0.002 | | 0.032 | | | 0.056 | |  |  |
| ***Verbal Reasoning*** |  |  |  |  |  |  | |  | | |  |  |  |  |  |
| Atheist vs. Agnostic | 3.199 | 0.001 | 0.018 | 0.008 | -12.093 | | <0.0001 | | 0.119 | | | <0.0001 | |  |  |
| Atheist vs. Religious | 13.270 | <0.0001 | 0.065 | <0.0001 | -14.254 | | <0.0001 | | 0.110 | | | <0.0001 | |  |  |
| Agnostic vs. Religious | 9.631 | <0.0001 | 0.050 | <0.0001 | 0.804 | | 0.422 | | 0.026 | | | 0.178 | |  |  |
| ***Overall Mean*** |  |  |  |  |  |  | |  | | |  |  |  |  |  |
| Atheist vs. Agnostic | 7.345 | <0.0001 | 0.033 | <0.0001 | -12.341 | | <0.0001 | | 0.107 | | | <0.0001 | |  |  |
| Atheist vs. Religious | 18.220 | <0.0001 | 0.090 | <0.0001 | -18.074 | | <0.0001 | | 0.128 | | | <0.0001 | |  |  |
| Agnostic vs. Religious | 10.511 | <0.0001 | 0.612 | <0.0001 | -1.783 | | 0.075 | | 0.033 | | | 0.049 | |  |  |

Supplementary table 11. Pairwise comparisons between the religious sub groups (Study 2) component scores. T-tests statistics (t) were calculated to assess whether means differed between groups & kolmogorov-Sminov (ks) statistics were calculated to assess whether the two samples came from independent distributions.

| ***Study 2 - Religious sub groups*** | | | |  | |  | |  | |  |  | |  | |  | | |
| --- | --- | --- | --- | --- | --- | --- | --- | --- | --- | --- | --- | --- | --- | --- | --- | --- | --- |
|  | | t | p | | ks | | p | | t | | p | ks | | p | |  |  |
|  | | ***Reasoning*** | | | | | | | ***Working Memory*** | | | | | | | |  |
| R1 vs. Agnostic | -8.411 | | <0.0001 | | 0.081 | | <0.0001 | | -1.002 | | 0.317 | 0.016 | | 0.664 | |  |  |
| R1 vs. Atheist | | -11.586 | <0.0001 | | 0.091 | | <0.0001 | | -5.438 | | <0.0001 | 0.038 | | 0.000 | |  |  |
| R1 vs. R3 | | 2.609 | 0.009 | | 0.084 | | 0.007 | | -0.304 | | 0.761 | 0.037 | | 0.652 | |  |  |
| R1 vs. R4 | | -0.874 | 0.382 | | 0.038 | | 0.652 | | 1.863 | | 0.063 | 0.086 | | 0.007 | |  |  |
| R1 vs. R5 | | 0.941 | 0.347 | | 0.045 | | 0.459 | | 0.242 | | 0.809 | 0.055 | | 0.238 | |  |  |
| R1 vs. R2 | | -2.112 | 0.035 | | 0.078 | | 0.008 | | -1.086 | | 0.278 | 0.038 | | 0.519 | |  |  |
| Agnostic vs. Atheist | | -0.762 | 0.446 | | 0.024 | | 0.295 | | -3.126 | | 0.002 | 0.032 | | 0.056 | |  |  |
| Agnostic vs. R3 | | 5.871 | <0.0001 | | 0.144 | | <0.0001 | | 0.132 | | 0.895 | 0.028 | | 0.947 | |  |  |
| Agnostic vs. R4 | | 2.558 | 0.011 | | 0.102 | | 0.001 | | 2.271 | | 0.023 | 0.089 | | 0.008 | |  |  |
| Agnostic vs. R5 | | 4.178 | <0.0001 | | 0.116 | | 0.000 | | 0.655 | | 0.512 | 0.052 | | 0.334 | |  |  |
| Agnostic vs. R2 | | 1.656 | 0.098 | | 0.061 | | 0.098 | | -0.613 | | 0.540 | 0.037 | | 0.633 | |  |  |
| Atheist vs. R3 | | 6.374 | <0.0001 | | 0.151 | | <0.0001 | | 1.584 | | 0.113 | 0.049 | | 0.310 | |  |  |
| Atheist vs. R4 | | 2.970 | 0.003 | | 0.110 | | 0.000 | | 3.688 | | 0.000 | 0.116 | | <0.0001 | |  |  |
| Atheist vs. R5 | | 4.599 | <0.0001 | | 0.123 | | <0.0001 | | 2.026 | | 0.043 | 0.082 | | 0.019 | |  |  |
| Atheist vs. R2 | | 2.102 | 0.036 | | 0.058 | | 0.106 | | 0.930 | | 0.352 | 0.043 | | 0.391 | |  |  |
| R3 vs. R4 | | -2.364 | 0.018 | | 0.090 | | 0.074 | | 1.545 | | 0.123 | 0.080 | | 0.146 | |  |  |
| R3 vs. R5 | | -1.101 | 0.271 | | 0.080 | | 0.164 | | 0.396 | | 0.692 | 0.066 | | 0.358 | |  |  |
| R3 vs. R2 | | -3.022 | 0.003 | | 0.115 | | 0.005 | | -0.535 | | 0.593 | 0.047 | | 0.699 | |  |  |
| R4 vs. R5 | | 1.253 | 0.210 | | 0.044 | | 0.857 | | -1.086 | | 0.278 | 0.050 | | 0.723 | |  |  |
| R4 vs. R2 | | -0.703 | 0.482 | | 0.083 | | 0.100 | | -2.057 | | 0.040 | 0.120 | | 0.004 | |  |  |
| R5 vs. R2 | | -1.912 | 0.056 | | 0.099 | | 0.031 | | -0.900 | | 0.368 | 0.083 | | 0.111 | |  |  |
| ***Verbal Reasoning*** | | | | | | | ***Overall Mean*** | | | | | | | |  |  |  |
| R1 vs. Agnostic | | -12.195 | <0.0001 | | 0.124 | | <0.0001 | | -12.217 | | <0.0001 | 0.108 | | <0.0001 | |  |  |
| R1 vs. Atheist | | -14.225 | <0.0001 | | 0.114 | | <0.0001 | | -17.677 | | <0.0001 | 0.129 | | <0.0001 | |  |  |
| R1 vs. R3 | | 3.158 | 0.002 | | 0.072 | | 0.032 | | 3.068 | | 0.002 | 0.086 | | 0.005 | |  |  |
| R1 vs. R4 | | -4.499 | <0.0001 | | 0.108 | | 0.000 | | -1.962 | | 0.050 | 0.057 | | 0.166 | |  |  |
| R1 vs. R5 | | 0.603 | 0.547 | | 0.039 | | 0.658 | | 1.009 | | 0.313 | 0.054 | | 0.245 | |  |  |
| R1 vs. R2 | | -1.673 | 0.094 | | 0.053 | | 0.159 | | -2.768 | | 0.006 | 0.085 | | 0.003 | |  |  |
| Agnostic vs. Atheist | | 0.804 | 0.422 | | 0.026 | | 0.178 | | -1.783 | | 0.075 | 0.033 | | 0.049 | |  |  |
| Agnostic vs. R3 | | 8.378 | <0.0001 | | 0.177 | | <0.0001 | | 8.213 | | <0.0001 | 0.189 | | <0.0001 | |  |  |
| Agnostic vs. R4 | | 0.683 | 0.495 | | 0.061 | | 0.157 | | 3.172 | | 0.002 | 0.100 | | 0.002 | |  |  |
| Agnostic vs. R5 | | 5.542 | <0.0001 | | 0.130 | | <0.0001 | | 6.009 | | <0.0001 | 0.156 | | <0.0001 | |  |  |
| Agnostic vs. R2 | | 3.929 | <0.0001 | | 0.104 | | 0.000 | | 2.832 | | 0.005 | 0.082 | | 0.008 | |  |  |
| Atheist vs. R3 | | 8.086 | <0.0001 | | 0.177 | | <0.0001 | | 9.031 | | <0.0001 | 0.200 | | <0.0001 | |  |  |
| Atheist vs. R4 | | 0.322 | 0.747 | | 0.038 | | 0.651 | | 3.985 | | <0.0001 | 0.108 | | 0.000 | |  |  |
| Atheist vs. R5 | | 5.253 | <0.0001 | | 0.112 | | 0.000 | | 6.747 | | <0.0001 | 0.179 | | <0.0001 | |  |  |
| Atheist vs. R2 | | 3.596 | 0.000 | | 0.109 | | <0.0001 | | 3.742 | | 0.000 | 0.085 | | 0.003 | |  |  |
| R3 vs. R4 | | -5.453 | <0.0001 | | 0.165 | | <0.0001 | | -3.625 | | 0.000 | 0.110 | | 0.015 | |  |  |
| R3 vs. R5 | | -1.679 | 0.094 | | 0.089 | | 0.085 | | -1.497 | | 0.135 | 0.097 | | 0.049 | |  |  |
| R3 vs. R2 | | -3.455 | 0.001 | | 0.106 | | 0.013 | | -4.186 | | <0.0001 | 0.149 | | <0.0001 | |  |  |
| R4 vs. R5 | | 3.438 | 0.001 | | 0.111 | | 0.017 | | 2.199 | | 0.028 | 0.102 | | 0.037 | |  |  |
| R4 vs. R2 | | 2.190 | 0.029 | | 0.107 | | 0.013 | | -0.414 | | 0.679 | 0.057 | | 0.483 | |  |  |
| R5 vs. R2 | | -1.506 | 0.133 | | 0.062 | | 0.392 | | -2.691 | | 0.007 | 0.124 | | 0.003 | |  |  |

Supplementary table 12. Component scores for each group showing distance to the population mean in Standard Deviation units (mean 0, SD 1).

|  | Reasoning | Working Memory | Verbal Reasoning | Overall Mean |
| --- | --- | --- | --- | --- |
| ***Study 1*** |  |  |  |  |
| Atheist | 0.091 | 0.009 | 0.050 | 0.050 |
| Agnostic | 0.020 | -0.007 | 0.027 | 0.014 |
| Religious | -0.153 | -0.006 | -0.103 | -0.087 |
| ***Study 2*** |  | | | |
| Atheist | 0.082 | 0.063 | 0.118 | 0.175 |
| Agnostic | 0.070 | -0.008 | 0.133 | 0.136 |
| Religious | -0.046 | -0.023 | -0.073 | -0.096 |
| **Study 2 – Religious Dogmatism** | | | | |
| Absolute Certainty | -0.043 | -0.052 | -0.107 | -0.132 |
| Strong | -0.067 | -0.050 | -0.106 | -0.149 |
| Not Certain | -0.041 | -0.021 | -0.055 | -0.079 |
| Very Doubtful | 0.000 | -0.024 | 0.051 | 0.020 |
| Atheist | 0.065 | 0.058 | 0.080 | 0.134 |
| **Study 2 – Religious Subgroups** | | | | |
| R3 | -0.135 | -0.015 | -0.212 | -0.250 |
| R5 | -0.082 | -0.042 | -0.109 | -0.158 |
| R1 | -0.050 | -0.030 | -0.083 | -0.109 |
| R2 | 0.014 | 0.020 | -0.019 | 0.010 |
| R4 | -0.021 | -0.124 | 0.104 | -0.017 |
| Agnostic | 0.070 | -0.008 | 0.133 | 0.136 |
| Atheist | 0.082 | 0.063 | 0.118 | 0.175 |

Supplementary table 13. Main and interaction effects of Religious Group and Cognitive Task Performance.

| ***Religious Class & Group interacts with Cognitive task*** | | | |
| --- | --- | --- | --- |
| ***Study 1*** | df | F | p |
| Religious Class | 2 | 100.395 | <0.0001 |
| Task | 11 | 3.2e-07 | 0.949 |
| Religious Class * Task | 22 | 12.818 | <0.0001 |
| Error | 492547 |  |  |
| Total | 537359 |  |  |
| ***Study 2*** |  |  |  |
| Religious Class | 2 | 78.484 | <0.0001 |
| Task | 12 | 53.259 | <0.0001 |
| Religious Class * Task | 24 | 6.719 | <0.0001 |
| Error | 91848 |  |  |
| Total | 99540 |  |  |
| ***Study 2 - Religious Sub groups*** |  |  |  |
| Religious Group | 6 | 30.023 | <0.0001 |
| Task | 12 | 53.344 | <0.0001 |
| Religious Group * Task | 72 | 4.935 | <0.0001 |
| Error | 91800 |  |  |
| Total | 99540 |  |  |

Supplementary table 14. Task performances in Standard Deviation (SD) units for both Studies across the religious agnostic and atheist groups (Performance confounds Age, Education & Country of Origin factored out).

| **Study 1** | Religious | Agnostic | Atheist |  | **Study 2** | Religious | Agnostic | Atheist |
| --- | --- | --- | --- | --- | --- | --- | --- | --- |
| Grammatical Reasoning | -0.112 | 0.029 | 0.055 |  | Interlocking Polygons | -0.081 | 0.101 | 0.150 |
| Colour Word Remapping | -0.081 | -0.002 | 0.057 |  | Colour Word Remapping | -0.094 | 0.186 | 0.138 |
| Interlocking Polygons | -0.080 | 0.013 | 0.045 |  | Analogical Reasoning | -0.076 | 0.079 | 0.143 |
| Spatial Rotations | -0.062 | -0.017 | 0.056 |  | Grammatical Reasoning | -0.057 | 0.070 | 0.098 |
| Feature Match | -0.060 | -0.003 | 0.044 |  | Self Orderred Search | -0.056 | 0.085 | 0.098 |
| Self Orderred Search | -0.040 | -0.006 | 0.033 |  | Spatial Rotations | -0.046 | 0.037 | 0.098 |
| Spatial Span | -0.039 | -0.007 | 0.032 |  | Feature Match | -0.054 | 0.094 | 0.088 |
| Hampshire Tree | -0.036 | -0.001 | 0.026 |  | Moving Flanker | -0.048 | 0.055 | 0.072 |
| Digit Span | -0.035 | -0.003 | 0.026 |  | Spatial Chunking | -0.035 | 0.017 | 0.081 |
| Monkey Ladder | -0.012 | -0.009 | 0.015 |  | Hampshire Tree | -0.042 | 0.057 | 0.073 |
| Deductive Reasoning | -0.008 | -0.008 | 0.011 |  | Monkey Ladder | -0.033 | 0.020 | 0.073 |
| Paired Associates | 0.002 | -0.010 | 0.007 |  | Digit Span | -0.032 | 0.052 | 0.056 |
|  |  |  |  |  | Deductive Reasoning | -0.009 | 0.029 | 0.009 |

Supplementary table 15. Main and interaction effects between Religious Group and Age Group and between Religious Group and Level of Education on Overall Mean component score.

| **Age Group** | | | |  | | **Level of Education** | | | | | | | |  |
| --- | --- | --- | --- | --- | --- | --- | --- | --- | --- | --- | --- | --- | --- | --- |
| ***Study 1*** | df | F | p | |  | |  | | df | | F | | p | |
| Religious Group | 2 | 72.313 | <0.0001 | |  | | Religious Group | | 2 | | 119.088 | | <0.0001 | |
| Age Group | 5 | 25.265 | <0.0001 | |  | | Education | | 4 | | 0.091 | | 0.985 | |
| Religious Group * Age Group | 10 | 1.566 | 0.109 | |  | | Religious Group * Education | | 8 | | 0.987 | | 0.443 | |
| Error | 44347 |  |  | |  | | Error | | 44765 | |  | |  | |
| Total | 44364 |  |  | |  | | Total | | 44779 | |  | |  | |
| ***Study 2*** |  |  |  | |  | |  | |  | |  | |  | |
| Religious Group | 2 | 88.475 | <0.0001 | |  | | Religious Group | | 2 | | 180.974 | | <0.0001 | |
| Age Group | 5 | 37.966 | <0.0001 | |  | | Education | | 6 | | 0.417 | | 0.869 | |
| Religious Group * Age Group | 10 | 0.584 | 0.828 | |  | | Religious Group * Education | | 12 | | 1.325 | | 0.196 | |
| Error | 18402 |  |  | |  | | Error | | 18434 | |  | |  | |
| Total | 18419 |  |  | |  | | Total | | 18454 | |  | |  | |
| ***Study 2 - Religious Sub groups*** |  |  |  | |  | |  | |  | |  | |  | |
| Religious Group | 6 | 30.126 | <0.0001 | |  | | Religious Group | | 6 | | 64.689 | | <0.0001 | |
| Age Group | 5 | 2.427 | 0.033 | |  | | Education | | 6 | | 0.991 | | 0.429 | |
| Religious Group * Age Group | 30 | 1.223 | 0.187 | |  | | Religious Group * Education | | 36 | | 1.257 | | 0.139 | |
| Error | 18378 |  |  | |  | | Error | | 18406 | |  | |  | |
| Total | 18419 |  |  | |  | | Total | | 18454 | |  | |  | |
| **Country of Origin** | | | |  | |  | |  | |  | |  | |  |
| ***Study 1*** | df | F | p | |  | |  | |  | |  | |  | |
| Religious Group | 2 | 78.642 | <0.0001 | | | |  | |  | |  | |  | |
| Country | 9 | 1.227 | 0.272 | |  | |  | |  | |  | |  | |
| Religious Group * Country | 18 | 2.853 | <0.0001 | | | |  | |  | |  | |  | |
| Error | 44750 |  |  | |  | |  | |  | |  | |  | |
| Total | 44779 |  |  | |  | |  | |  | |  | |  | |
| ***Study 2*** |  |  |  | |  | |  | |  | |  | |  | |
| Religious Group | 2 | 42.224 | <0.0001 | | | |  | |  | |  | |  | |
| Country | 8 | 1.45 | 0.17 | |  | |  | |  | |  | |  | |
| Religious Group * Country | 16 | 2.035 | 0.009 | |  | |  | |  | |  | |  | |
| Error | 18428 |  |  | |  | |  | |  | |  | |  | |
| Total | 18454 |  |  | |  | |  | |  | |  | |  | |
| ***Study 2 - Religious subgroups*** |  |  |  | |  | |  | |  | |  | |  | |
| Religious Group | 6 | 12.959 | <0.0001 | | | |  | |  | |  | |  | |
| Country | 8 | 1.716 | 0.089 | |  | |  | |  | |  | |  | |
| Religious Group * Country | 48 | 1.659 | 0.003 | |  | |  | |  | |  | |  | |
| Error | 18392 |  |  | |  | |  | |  | |  | |  | |
| Total | 18454 |  |  | |  | |  | |  | |  | |  | |

Supplementary table 16. Main effect of Individual Dogma on Components of Cognition (Study 2).

| **Study 2** | df | F | p |
| --- | --- | --- | --- |
| ***Reasoning*** |  |  |  |
| Individual Dogma | 4 | 30.838 | p<0.0001 |
| Error | 19518 |  |  |
| Total | 19522 |  |  |
| ***Working Memory*** |  |  |  |
| Individual Dogma | 4 | 10.485 | p<0.0001 |
| Error | 19518 |  |  |
| Total | 19522 |  |  |
| ***Verbal Reasoning*** |  |  |  |
| Individual Dogma | 4 | 46.010 | p<0.0001 |
| Error | 19518 |  |  |
| Total | 19522 |  |  |
| ***Overall Mean*** |  |  |  |
| Individual Dogma | 4 | 74.774 | p<0.0001 |
| Error | 19518 |  |  |
| Total | 19522 |  |  |

Supplementary table 17. Pairwise comparisons of component scores between the levels of Individual Dogma (Study 2). T-tests statistics (t) were calculated to assess whether means differed between groups & kolmogorov-Sminov (ks) statistics were calculated to assess whether the two samples came from independent distributions.

|  | t | | p | ks | p |  | t | p | ks | p | |
| --- | --- | --- | --- | --- | --- | --- | --- | --- | --- | --- | --- |
| ***Reasoning*** | |  |  |  |  | ***Working Memory*** | | | | |  |
| Absolute Certainty vs. Strong | | 1.183 | 0.237 | 0.022 | 0.700 |  | -0.080 | 0.936 | 0.020 | 0.807 | |
| Absolute Certainty vs. Not Certain | | -0.139 | 0.890 | 0.020 | 0.759 |  | -1.057 | 0.291 | 0.024 | 0.505 | |
| Absolute Certainty vs. Very Doubtful | | -2.183 | 0.029 | 0.039 | 0.077 |  | -0.984 | 0.325 | 0.031 | 0.259 | |
| Absolute Certainty vs. Atheist | | -5.820 | <0.0001 | 0.074 | <0.0001 |  | -4.043 | <0.0001 | 0.054 | 0.001 | |
| Strong vs. Not Certain | | -1.721 | 0.085 | 0.028 | 0.124 |  | -1.245 | 0.213 | 0.020 | 0.442 | |
| Strong vs. Very Doubtful | | -4.201 | <0.0001 | 0.046 | 0.002 |  | -1.112 | 0.266 | 0.033 | 0.049 | |
| Strong vs. Atheist | | -9.314 | <0.0001 | 0.080 | <0.0001 |  | -5.176 | <0.0001 | 0.051 | <0.0001 | |
| Not certain vs. Very Doubtful | | -2.739 | 0.006 | 0.028 | 0.099 |  | 0.133 | 0.894 | 0.020 | 0.410 | |
| Not Certain vs. Atheist | | -8.282 | <0.0001 | 0.063 | <0.0001 |  | -4.193 | <0.0001 | 0.039 | 0.001 | |
| Very Doubtful vs. Atheist | | -4.709 | <0.0001 | 0.047 | <0.0001 |  | -4.154 | <0.0001 | 0.037 | 0.003 | |
| ***Verbal Reasoning*** | |  |  |  |  | ***Overall Mean*** | | | | |  |
| Absolute Certainty vs. Strong | | -0.025 | 0.980 | 0.020 | 0.789 |  | 0.575 | 0.565 | 0.024 | 0.584 | |
| Absolute Certainty vs. Not Certain | | -2.131 | 0.033 | 0.033 | 0.162 |  | -1.968 | 0.049 | 0.036 | 0.100 | |
| Absolute Certainty vs. Very Doubtful | | -6.545 | <0.0001 | 0.087 | <0.0001 |  | -5.475 | <0.0001 | 0.077 | <0.0001 | |
| Absolute Certainty vs. Atheist | | -8.262 | <0.0001 | 0.097 | <0.0001 |  | -10.214 | <0.0001 | 0.118 | <0.0001 | |
| Strong vs. Not Certain | | -2.655 | 0.008 | 0.034 | 0.030 |  | -3.273 | 0.001 | 0.046 | 0.001 | |
| Strong vs. Very Doubtful | | -7.978 | <0.0001 | 0.085 | <0.0001 |  | -7.543 | <0.0001 | 0.088 | <0.0001 | |
| Strong vs. Atheist | | -10.692 | <0.0001 | 0.099 | <0.0001 |  | -14.227 | <0.0001 | 0.129 | <0.0001 | |
| Not certain vs. Very Doubtful | | -5.827 | <0.0001 | 0.063 | <0.0001 |  | -4.866 | <0.0001 | 0.051 | <0.0001 | |
| Not Certain vs. Atheist | | -8.685 | <0.0001 | 0.074 | <0.0001 |  | -12.139 | <0.0001 | 0.096 | <0.0001 | |
| Very Doubtful vs. Atheist | | -1.762 | 0.078 | 0.022 | 0.195 |  | -6.033 | <0.0001 | 0.050 | <0.0001 | |

Supplementary table 18. Task scores across levels of Individual Dogmatism in Standard Deviation units (mean=0, SD=1; Study 2).

|  | Absolute Certainty | Strong | Not Certain | Very Doubtful | Atheist |
| --- | --- | --- | --- | --- | --- |
| Monkey Ladder | -0.107 | -0.070 | -0.024 | -0.032 | 0.085 |
| Interlocking Polygons | -0.114 | -0.165 | -0.084 | 0.027 | 0.128 |
| Grammatical Reasoning | -0.125 | -0.127 | -0.057 | 0.022 | 0.105 |
| Colour Word Remapping | -0.093 | -0.107 | -0.079 | 0.033 | 0.107 |
| Deductive Reasoning | -0.035 | -0.030 | -0.005 | -0.009 | 0.021 |
| Self Ordered Search | -0.100 | -0.123 | -0.045 | 0.022 | 0.104 |
| Spatial Rotations | -0.158 | -0.135 | -0.056 | 0.012 | 0.116 |
| Feature Match | -0.141 | -0.150 | -0.041 | 0.008 | 0.115 |
| Digit Span | -0.103 | -0.065 | 0.019 | 0.030 | 0.029 |
| Hampshire Tree | -0.102 | -0.143 | -0.059 | 0.016 | 0.110 |
| Spatial Chunking | -0.102 | -0.092 | -0.042 | -0.025 | 0.094 |
| Moving Flanker | -0.035 | -0.067 | -0.061 | 0.007 | 0.069 |

Supplementary table 19. Task scores across religious and non-religious groups in Standard Deviation units (mean=0, SD=1; Study 2).

|  | R3 | R5 | R1 | R2 | R4 | Agnostic | Atheist |
| --- | --- | --- | --- | --- | --- | --- | --- |
| Monkey Ladder | -0.047 | -0.020 | -0.086 | 0.172 | -0.119 | 0.056 | 0.111 |
| Interlocking Polygons | -0.285 | -0.354 | -0.123 | 0.011 | -0.114 | 0.145 | 0.204 |
| Grammatical Reasoning | -0.206 | -0.120 | -0.117 | 0.131 | 0.085 | 0.129 | 0.151 |
| Colour Word Remapping | -0.429 | -0.157 | -0.130 | -0.112 | 0.171 | 0.264 | 0.184 |
| Deductive Reasoning | -0.044 | -0.147 | -0.032 | 0.033 | -0.032 | 0.052 | 0.036 |
| Self Ordered Search | -0.195 | -0.257 | -0.077 | -0.085 | -0.082 | 0.146 | 0.152 |
| Spatial Rotations | -0.339 | -0.452 | -0.074 | -0.045 | -0.034 | 0.089 | 0.156 |
| Feature Match | -0.239 | -0.347 | -0.104 | 0.113 | -0.040 | 0.146 | 0.152 |
| Digit Span | -0.101 | 0.295 | -0.091 | 0.239 | 0.221 | 0.064 | 0.064 |
| Hampshire Tree | -0.214 | -0.349 | -0.085 | 0.084 | -0.077 | 0.105 | 0.127 |
| Spatial Chunking | 0.031 | 0.115 | -0.100 | 0.192 | -0.186 | 0.043 | 0.120 |
| Moving Flanker | -0.187 | -0.296 | -0.083 | -0.014 | 0.119 | 0.100 | 0.113 |
| Analogical Reasoning | -0.217 | -0.233 | -0.125 | 0.048 | 0.041 | 0.128 | 0.203 |
