# Supplementary material for: The Negative Relationship between Reasoning and Religiosity Is Underpinned by a Bias for Intuitive Responses Specifically When Intuition and Logic Are in Conflict
Source: Front Psychol. 2017 Dec 19;8:2191. doi: 10.3389/fpsyg.2017.02191 (PMC5742220; doi:10.3389/fpsyg.2017.02191)
Supplement: Supplementary file 2 [file DataSheet1.docx]

***Supplementary Materials***

A big data perspective reveals that learnt behavioural biases toward intuitive responses when intuition and logic are conflicted underpins the negative relationship between reasoning and religiosity

Richard E. Daws^1^ & Adam Hampshire^1*^

*Corresponding Author: [a.hampshire@imperial.ac.uk](mailto:a.hampshire@imperial.ac.uk)

^1^The Computational, Cognitive & Clinical Neuroimaging Laboratory (C^3^NL), Imperial College London, London, UK.

1. **Sociodemographic data**

It is important to note that when analysing data with very large N effects of negligible scale often have very low p values due to high statistical power. Therefore, we report a high-level overview of the sociodemographic analyses and invite the reader to examine the full statistics in the supplementary tables. All p values are < 0.001 unless otherwise stated.

We initially investigated the sociodemographic variables of both cohorts and identified age, education and country of origin as confounding variables to be controlled for in the following analyses of cognitive performance. We applied Analysis of Variance (ANOVA) and pairwise t-test and Kolmogorov-Smirnov tests (ks-test) to interrogate age across the religious and non-religious (agnostic & atheist) groups. The education and country of origin variables were assessed using χ^2^ test of independence.

A one-way ANOVA with Group (religious, agnostic, atheist) as the between subject factor and age as the dependent variable showed significant main effects of Group in both cohorts (Supplementary table 9). Pairwise comparisons (Supplementary table 2) showed that the religious groups mean age was significantly higher than the agnostic and atheist groups in both cohorts. Mean age did not differ between the atheist and agnostic groups in cohort 1; however, the ks-test showed that the samples came from independent distributions. Conversely, atheist mean age was significantly lower than the agnostic group in cohort 2; however, the ks-test showed no evidence that the distributions came from independent distributions. Taken with a simple comparison of the groups mean ages (Supplementary table 1) the statistically significant differences in age between the religious and non-religious groups was of a very small effect size (Cohort 1 = 0.007 SDs; Cohort 2 = 0.03 SDs).

The education variable was assessed using χ^2^ test of independence. Both cohorts showed a significant interaction between religious group and education level (see Supplementary table 3 for χ^2^ statistics). The largest difference in proportion between the groups were in College Graduates (religious = 31.5%, agnostic = 31.1%, atheist = 28.5%) for cohort 1 and in University (Graduates religious = 26.2%, agnostic = 25.7%, atheist = 31%) for cohort 2 (see Supplementary table 4).

The country of origin variable was summarised into global regions and assessed using χ^2^ test of independence. Both cohorts showed a significant interaction between religious group and global region (see Supplementary table 3 for χ^2^ statistics). The largest difference in proportions between the groups were in the USA (cohort 1: religious = 39.8%, agnostic = 41.7%, atheist = 31.9%; cohort 2: religious = 41.5%, agnostic = 50.9%, atheist = 41.5%) and UK (cohort 1: religious = 15.5%, agnostic = 19.6%, atheist = 27.3%) regions (see Supplementary table 5).

The analysis of the demographic variables demonstrated negligibly scaled but statistically significant relationships of religious group with age, country of origin and education. Therefore, these were factored out prior to the analysis of religiosity effects.
