# Supplementary material for: The Negative Relationship between Reasoning and Religiosity Is Underpinned by a Bias for Intuitive Responses Specifically When Intuition and Logic Are in Conflict
Source: Front Psychol. 2017 Dec 19;8:2191. doi: 10.3389/fpsyg.2017.02191 (PMC5742220; doi:10.3389/fpsyg.2017.02191)
Supplement: Supplementary file 3 [file DataSheet2.docx]

***Supplementary Materials***

A big data perspective reveals that learnt behavioural biases toward intuitive responses when intuition and logic are conflicted underpins the negative relationship between reasoning and religiosity

Richard E. Daws^1^ & Adam Hampshire^1*^

*Corresponding Author: [a.hampshire@imperial.ac.uk](mailto:a.hampshire@imperial.ac.uk)

^1^The Computational, Cognitive & Clinical Neuroimaging Laboratory (C^3^NL), Imperial College London, London, UK.

1. **Description of tasks**

Brief descriptions of the 12 tests used in study 1 (based on classical paradigms from the cognitive psychology literature).

Spatial Span is based on the Corsi Block Tapping Task (Corsi, 1972), a classical tool for measuring spatial short-term memory capacity. 16 squares are displayed in a 4 * 4 grid. A sub-set of the squares flash in a random sequence at a rate of 1 flash every 900 ms. Subsequently, the mouse cursor is displayed and a tone cues the participant to repeat the sequence by clicking on the squares in the same order in which they flashed. The test starts with four flashes and difficulty on subsequent trials is dynamically varied. If the participant responds correctly, the length of the next sequence increases by one flash, otherwise the length of the next sequence is one flash shorter. The test finishes after 3 errors. Maximum level = 16, minimum level = 2. Outcome measure = maximum level achieved. Population mean = 6.15, standard deviation (SD) = 1.07.

Spatial chunking is a variant on the Spatial Span task. It includes sequences of stimuli that can be easily formed into geometric chunks, which can aid working memory performance. Outcome measure = maximum level achieved for non-geometric sequences plus the maximum level achieved for geometric sequences. Population mean = 11.13, standard deviation (SD) = 3.11.

Visuospatial Working Memory is based on a task from the non-human primate literature (Inoue and Matsuzawa, 2007). Sets of numbered squares are displayed all at the same time at random locations within an invisible 5*5 grid. After a variable interval (number of squares * 900 ms), the numbers are removed leaving just the blank squares visible and a tone cues the participant to respond by clicking on the squares in ascending numerical sequence. The level of difficulty is dynamically varied, starting with just 2 numbered squares on the first trial and then increasing or decreasing by 1 depending on whether or not the response is correct. The test finishes after three errors. Maximum level = 25 and minimum level = 2. Outcome measure = maximum level achieved. Population mean = 7.85, SD = 1.154.

Self-Ordered Search is based on a test that is widely used to measure strategy during search behaviour (Collins et al., 1998). Sets of boxes are displayed on the screen in random locations within an invisible 5*5 grid. The participant must find a hidden „token‟ by clicking on the boxes one at a time to reveal their contents. When the token is found, it is hidden within another box. On any given trial, the token will not appear within the same box twice, thus, the participant must search the boxes until the token has been found once within each box. If they search the same empty box twice whilst looking for the token, or search a box in which the token has previously been found, this is an error and the trial ends, subsequent to which a new trial begins with one less box to search. If the token is found once in each box without any errors being made, a new trial begins with one extra box to search. After three errors the test ends. The test starts with just four boxes. Maximum level = 25 and minimum level = 2. Outcome measure = maximum level achieved. Population mean = 8.23, SD = 2.10.

Paired Associates is based on a paradigm that is commonly used to assess memory impairments in aging clinical populations (Gould et al., 2005). Boxes are displayed at random locations on an invisible 5*5 grid. The boxes open one after another to reveal an enclosed object. Subsequently, the objects are displayed in random order in the centre of the grid and the participant must click on the boxes that contained them. If the participant remembers all of the object-location paired associates correctly then the difficulty level on the subsequent trial increases by 1 object-box pair, otherwise it decreases by 1. After three errors the test ends. The test starts with just two boxes. Maximum level = 24 minimum level = 2. Outcome measure = maximum level achieved. Population mean = 5.28, SD = 1.13.

Spatial Planning is based on the Tower of London Task (Shallice, 1982), which is widely used to measure executive function. Numbered beads are positioned on a tree shaped frame. The participant repositions the beads so that they are configured in ascending numerical order running from left to right and top to bottom of the tree. To gain maximum points, the participant must solve as many problems as possible in as few moves as possible within 3 minutes. Problems become progressively harder with the total number of moves required and the planning complexity increasing in steps. Trials are aborted if the participant makes more than twice the number of moves required to solve the problem. After each trial, the total score is incremented by adding the minimum number of moves required * 2 – the number of moves actually made, thereby rewarding efficient planning. The first problem can be solved in just 3 moves. Outcome measures = total score. Population mean = 64 SD = 10.185.

Spatial Rotation tasks are often used for measuring the ability to manipulate objects spatially in mind (Silverman et al., 2000). In this variant, two grids of colored squares are displayed to either side of the screen with one of the grids rotated by a multiple of 90 degrees. When rotated, the grids are either identical or differ by the position of just one square. In order to gain maximum points, the participant must indicate whether the grids are identical, solving as many problems as possible within 90 seconds. If the response is correct, the total score increases by the number of squares in the grid and subsequent trials have more squares. If the response is incorrect the total score decreases by the number of squares in the grid and subsequent trials have fewer squares. The first grids contain 4 colored squares each. Outcome measure = total score. Population mean = 88.72, SD = 36.32.

Feature Match is based on the classic feature search tasks that have historically been used to measure attentional processing (Treisman and Gelade, 1980). Two grids are displayed on the screen, each containing a set of abstract shapes. In half of the trials the grids differ by just one shape. In order to gain maximum points, the participant must indicate whether or not the grid‟s contents are identical, solving as many problems as possible within 90 seconds. If the response is correct, the total score increases by the number of shapes in the grid and the number of shapes in subsequent trials increases. If the response is incorrect the total score decreases by the number of shapes in the grid and subsequent trials have fewer shapes. The first grids contain two abstract shapes each. Outcome measure = total score. Population mean = 131.35, SD = 32.79.

Interlocking Polygons is based on the Interlocking Pentagons task, which is often used in the assessment of age related disorders (Folstein et al., 1975). A pair of overlapping polygons is displayed on one side of the screen. In order to gain maximum points, the participant must indicate whether a polygon displayed on the other side of the screen is identical to one of the interlocking polygons, solving as many problems as possible within 90 seconds. If responses are correct, the total score increases by the difficulty level and the differences between the polygons becomes increasingly subtle. If the responses are incorrect the total score decreases by the difficulty level and the difference between the polygons become more pronounced. Main outcome measure = total score. Population mean = 51.41, SD = 24.86.

Deductive Reasoning is based on a sub-set of problems from the Cattell Culture Fair Intelligence Test (Cattell, 1949). A 3* 3 grid of cells is displayed on the screen. Each cell contains a variable number of copies of a colored shape. The features that make up the objects in each cell (color, shape, number of copies) are related to each other according to a set of rules. The participant must deduce the rules that relate the object features and select the one cell whose contents do not correspond to those rules. To gain maximum points, the participant must solve as many problems as possible within 90 seconds. If the response is correct, the total score increases by one point and the next problem is more complex. If the response is incorrect, the total score decreases by 1 point. Outcome measure = total correct. Population mean = 10.43, SD = 3.31.

Digit Span is a computerised variant on the verbal working memory component of the WAIS-R intelligence test (Weschler, 1981). Participants view a sequence of digits that appear on the screen one after another. Subsequently, they repeat the sequence of numbers by entering them on the keyboard. Difficulty is dynamically varied with the number of digits to remember increasing or decreasing by 1 depending on whether the participant got the previous trial correct. The test ends after 3 errors. Maximum level = 25 and minimum level = 2. Outcome measure = maximum level achieved. Population mean = 7.22, SD = 1.52.

Verbal Reasoning is based on Alan Baddeley‟s 3 minute grammatical reasoning test (Baddeley, 1968). Problems of the form “The square is not encapsulated by the circle” are displayed on the screen and the participant must indicate whether the statement correctly describes a pair of objects displayed in the centre of the screen. In order to achieve maximum points, the participant must solve as many problems as possible within 90 seconds. Total score increases or decreases by 1 after each trial depending on whether responses are correct. The first trial has four numbers. The maximum level is 25 and the minimum level is 2. Outcome measure = total score. Population mean = 17.38, SD = 5.01.

Color-Word Remapping is a more challenging variant on the Stroop test (Stroop, 1935). A colored word is displayed at the top of the screen, for example the word RED drawn in blue ink. The participants must indicate which of two colored words at the bottom of the screen describes the color that the word at the top of the screen is drawn in. The color word mappings may be congruent, incongruent, or doubly incongruent, depending on whether or not the colors that a given words describes matches the color that it is drawn in. To gain maximum points, the participant must solve as many problems as possible within 90 seconds. The total score increases or decreases by 1 after each trial depending on whether they responded correctly. Outcome measure = total score. Population mean = 30.92, SD = 13.01.

Moving Flanker requires participants whenever they see a target digit appear within a moving grid of numbers. The target may also appear in other positions on the grid, but these are distractors and must be ignored. Participants are required to respond as quickly and accurately as possible. Total score increases or decreases by 1 dependent on whether they detect or miss a target. The task runs for 2.5 minutes. Outcome measure = total score. Population mean = 15.41, SD = 6.57.

Analogical reasoning is a visual reasoning task that requires rules to be mapped from one visual dimension to another. Individuals were required to complete as many problems as they could within 3 minutes. Stimuli for this task have been reported previously (Hampshire et al. Lateral prefrontal cortex sub-regions make dissociable contributions during fluid reasoning. Cerebral Cortex. 2010). Outcome measure = total score. Population mean = 17.6, SD = 9.21.
